# Tribovoltaic Effect: Origin, Interface, Characteristic, Mechanism & Application

**DOI:** 10.1002/advs.202305460

**Published:** 2024-02-14

**Authors:** Zhi Zhang, Likun Gong, Ruifei Luan, Yuan Feng, Jie Cao, Chi Zhang

**Affiliations:** ^1^ CAS Center for Excellence in Nanoscience, Beijing Key Laboratory of Micro‐nano Energy and Sensor, Beijing Institute of Nanoenergy and Nanosystems Chinese Academy of Sciences Beijing 101400 P. R. China; ^2^ School of Nanoscience and Engineering University of Chinese Academy of Sciences Beijing 100049 P. R. China; ^3^ Center on Nanoenergy Research School of Physical Science and Technology Guangxi University Nanning 530004 P. R. China; ^4^ Institute of Intelligent Flexible Mechatronics Jiangsu University Zhenjiang 212013 P. R. China

**Keywords:** direct current, nanogenerator, self‐powered system, semiconductor interface, tribovoltaic effect

## Abstract

Tribovoltaic effect is a phenomenon of the generation of direct voltage and current by the mechanical friction on semiconductor interface, which exhibits a brand‐new energy conversion mechanism by the coupling of semiconductor and triboelectrification. Here, the origin, interfaces, characteristics, mechanism, coupling effect and application of the tribovoltaic effect is summarized and reviewed. The tribovoltaic effect is first proposed in 2019, which has developed in various forms tribovoltaic nanogenerator (TVNG) including metal‐semiconductor, metal‐insulator‐semiconductor, semiconductor‐semiconductor, liquid‐solid and flexible interfaces. Compared with triboelectric nanogenerator, the TVNG has the characteristics of direct‐current, high current density (mA‐A cm^−2^) and low impedance (Ω‐kΩ). The two mainstream views on the tribovoltaic generation mechanism, one dominated by built‐in electric fields and the other dominated by interface electric fields, have been elaborated and summarized in detail. The tribo‐photovoltaic effect and tribo‐thermoelectric effect are also discovered and introduced because they can easily interact with other multi‐physical field effects. The TVNGs are suitable for making energy harvesting and self‐powered sensing devices for micro‐nano energy applications. This paper not only revisit the development of the tribovoltaic effect, but also makes prospects for mechanism research, device fabrication and integrated application, which can accelerate the evolution of smart wearable electronics and intelligent industrial components.

## Graphical abstract

This review sheds light on the origin, current status, challenges, and prospects of the tribovoltaic effect on semiconductor interface. It can realize the efficient recovery and utilization of friction energy, promote the development of the Internet of Things era, and become an important support for energy saving and carbon reduction under the background of carbon neutrality.

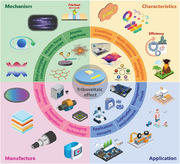

## 1 Introduction

### 1.1 The History of Semiconductor Effect

**Figure 1:**
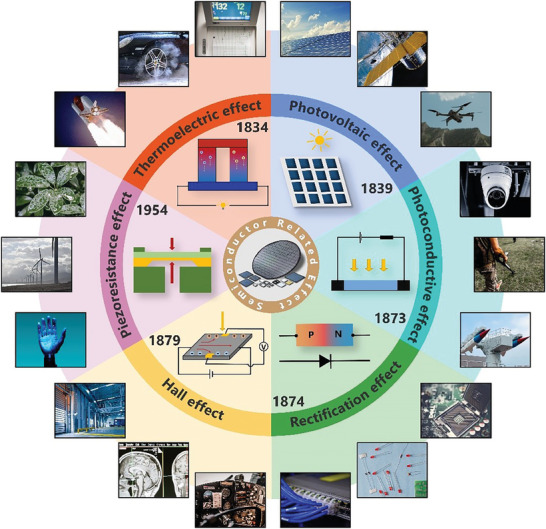
The history of semiconductor effect.

Semiconductor science and technology have brought a tremendous innovation in the development of human society, which is now widely applied in integrated circuits, consumer electronics, communication systems, photovoltaic power generation, lighting applications, high power supply conversion and other fields. It has become a crucial symbol of economic development, scientific and technological progress, and national defense strength. Over the past 180 years of history, many significant physical effects have been discovered based on semiconductors, which includes the exploration of semiconductor properties coupled with heat, light, electricity, magnetism and stress. A series of semiconductor effects, such as thermoelectricity (1834), photovoltaic (1839), photoconductivity (1873), rectification (1874), Hall (1879) and piezoresistance (1954) have been discovered (**Figure** **1**), which laid an important foundation for the development of semiconductor technology.^[^ ^1^, ^2^, ^3^, ^4^, ^5^, ^6^ ^]^

Based on study of the semiconductor effect, researchers have uncovered ways to harness micro‐nano energy from natural sources. For instance, thermoelectric generators can effectively recover waste heat from heat pipes utilizing semiconductors with high Seebeck coefficient.^[^ ^7^ ^]^ Additionally, solar cells can convert light into electrical energy for powering electronic devices by photovoltaic effect.^[^ ^8^ ^]^ The photoconductive effect enables photodiode to convert optical signals into electrical signals, widely employed in the sensing and monitoring applications.^[^ ^9^ ^]^ Semiconductor‐based rectifier devices play a crucial role in current and voltage regulation.^[^ ^10^ ^]^ Hall devices leverage a magnetic field as the working medium to convert the motion parameters of objects into a digital voltage output, imparting sensing and switching functions.^[^ ^11^ ^]^ Mechanical energy can be converted into electrical energy by piezoelectric effect, facilitating the harvesting of vibration energy in various environments and raindrop energy in ambient environment.^[^ ^12^, ^13^ ^]^ The multiphysics field semiconductor effects contribute to the conversion of diverse energy sources into electrical energy, significantly enhancing energy utilization efficiency and expanding the application scenarios of devices.

### 1.2 Frictional Energy Harvesting and Triboelectric Nanogenerator

The development of human society was inseparable from the use of advanced energy technologies. Surveys indicate that approximately one‐third of all human energy is consumed through friction, such as the friction energy consumption of basic components like bearings and gears in industrial equipment. If the recovery and utilization of friction energy can be achieved, energy conservation efficiency can be greatly improved, providing energy solutions for powering sensor network nodes.

In 2012, the creation of triboelectric nanogenerator (TENG) presented a novel approach for mechanical energy harvesting, capable of converting various environmental mechanical energy into electrical energy.^[^ ^14^ ^]^ Over the past decade, TENG has emerged as a global research hotspot in academia, with continuous improvements in its output performance through advancements in surface micro‐nano fabrication, material modification, and charge pumping methods.^[^ ^15^, ^16^, ^17^ ^]^ However, TENG is limited by the surface charge density of insulator. The key domain to overcome this challenge is the realization of volumetric TENG.^[^ ^18^, ^19^, ^20^ ^]^ Its typical characteristics of low current density (nA‐µA cm^−2^) and high impedance (MΩ‐GΩ) are difficult to make an essential breakthrough, which greatly limits the power density and technical applications of TENG. Urgent exploration of new principles is needed to break through performance bottlenecks and boost TENG toward practical engineering applications.

### 1.3 Proposal and Development of Tribovoltaic Effect

**Figure 2:**
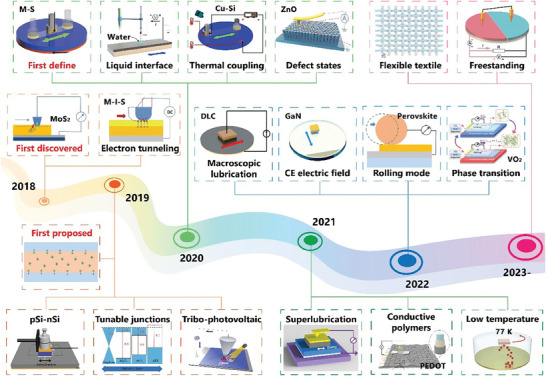
The development of tribovoltaic effect. Adapted from Liu et al.^[^ ^21^ ^]^ with permission. Copyright 2017, Nature Publishing Group. Adapted from Liu et al.^[^ ^22^ ^]^ with permission. Copyright 2018, Elsevier B.V. Adapted from Wang et al.^[^ ^23^ ^]^ with permission. Copyright 2019, Elsevier B.V. Adapted from Xu et al.^[^ ^24^ ^]^ with permission. Copyright 2019, Elsevier B.V. Adapted under terms of the CC‐BY license.^[^ ^25^ ^]^ Copyright 2019, Lu et al., published by American Association for the Advancement of Science. Adapted from Liu et al.^[^ ^26^ ^]^ with permission. Copyright 2019, Elsevier B.V. Adapted from Zhang et al.^[^ ^27^ ^]^ with permission. Copyright 2020, WILEY‐VCH Verlag GmbH & Co. KGaA, Weinheim. Adapted from Lin et al.^[^ ^29^ ^]^ with permission. Copyright 2020, Elsevier B.V. Adapted from Zhang et al.^[^ ^28^ ^]^ with permission. Copyright 2021, Elsevier B.V. Adapted from Song et al.^[^ ^30^ ^]^ with permission. Copyright 2021, Elsevier B.V. Adapted from Huang et al.^[^ ^32^ ^]^ with permission. Copyright 2021, Nature Publishing Group. Adapted from Meng et al.^[^ ^31^ ^]^ with permission. Copyright 2021, American Chemical Society. Adapted from Zheng et al.^[^ ^33^ ^]^ with permission. Copyright 2022, WILEY‐VCH Verlag GmbH & Co. KGaA, Weinheim. Adapted from Qiao et al.^[^ ^36^ ^]^ with permission. Copyright 2022, WILEY‐VCH Verlag GmbH & Co. KGaA, Weinheim. Adapted from Wang et al.^[^ ^35^ ^]^ with permission. Copyright 2022, The Royal Society of Chemistry. Adapted from Yuan et al.^[^ ^37^ ^]^ with permission. Copyright 2022, WILEY‐VCH Verlag GmbH & Co. KGaA, Weinheim. Adapted from Yang et al.^[^ ^38^ ^]^ with permission. Copyright 2022, American Chemical Society. Adapted under terms of the CC‐BY license.^[^ ^39^ ^]^ Copyright 2023, Deng et al., published by published by John Wiley and Sons. Adapted from Dong et al.^[^ ^40^ ^]^ with permission. Copyright 2023, WILEY‐VCH Verlag GmbH & Co. KGaA, Weinheim.

Since 2018, several research teams have utilized semiconductor materials to fabricate TENG, leading to the experimental observation of direct current (DC) electrical output, which is a phenomenon significantly distinct from traditional TENG (**Figure** **2**). In 2018, Liu et al. first observed the DC generation by sliding a metal probe on a molybdenum disulfide (MoS_2_) film.^[^ ^21^ ^]^ Concurrently, they identified the presence of electron tunneling existed in metal‐insulator‐semiconductor triboelectric interfaces.^[^ ^22^ ^]^ In 2019, Wang et al. first coined this phenomenon as “tribovoltaic effect”, which is similar to the photovoltaic effect.^[^ ^23^ ^]^ Xu, Lu, Lin et al. experimentally confirmed the tribovoltaic effect and discovered its photovoltaic coupling effect in Si interfaces.^[^ ^24^, ^25^, ^26^ ^]^ In 2020, Zhang et al. first formally defined the tribovoltaic effect as the generation of direct voltage and current by the mechanical friction on semiconductor interface. They simultaneously used the band theory and frictional excitation carrier to explain the tribovoltaic effect.^[^ ^27^ ^]^ Furthermore, they proposed a coupling power generation mechanism by combining the tribovoltaic effect with the thermoelectric effect.^[^ ^28^ ^]^ Lin et al. achieved DC power generation from liquid‐solid friction, providing a new idea for harvesting liquid movement energy.^[^ ^29^ ^]^ Song et al. observed the phenomenon of DC by the metal tip sliding on Zinc oxide (ZnO) nanowires, whose output is closely related to the concentration of oxygen vacancy defect states on the surface of ZnO nanowires.^[^ ^30^ ^]^ In 2021, Meng et al. implemented organic tribovoltaic nanogenerator (TVNG) based on flexible interfaces, broadening the development of TVNG in the wearable electronics.^[^ ^31^ ^]^ In the same year, Huang et al. investigated the structural superlubric TVNG and its output characteristics based on graphene with micro‐lamellar structure.^[^ ^32^ ^]^ Zheng et al. explored the output characteristics of TVNG under low temperature, noting an improvement in TVNG performance as the temperature decreased, which promoted the application in low‐temperature environments.^[^ ^33^ ^]^ In 2022, Zhang et al. first proposed the mechanism based on interfacial electric filed to explain tribovoltaic effect and realized the high performance TVNG with ultra‐high voltage and averaged power density.^[^ ^34^, ^35^ ^]^ Qiao et al. accomplished macroscopic lubrication using water‐based graphene, significantly enhancing improves the wear resistance of TVNG.^[^ ^36^ ^]^ Then Yuan et al. designed a rolling‐mode TVNG for harvesting mechanical and solar energy.^[^ ^37^ ^]^ Yang et al. realized the transition of Vanadium oxide from metal phase to semiconductor phase, realizing the controllable tuning of the output of the tribovoltaic effect.^[^ ^38^ ^]^ In 2023, Deng et al. fabricated tribovoltaic textiles using organic semiconductors, enabling distributed sensing through the preparation of tribovoltaic textiles.^[^ ^39^ ^]^ Dong et al. innovatively designed a freestanding‐mode TVNG (FTVNG), and seamlessly integrated FTVNG into rolling bearings to collect mechanical friction energy.^[^ ^40^ ^]^

### 1.4 Characteristics of Tribovoltaic Nanogenerator

**Figure 3:**
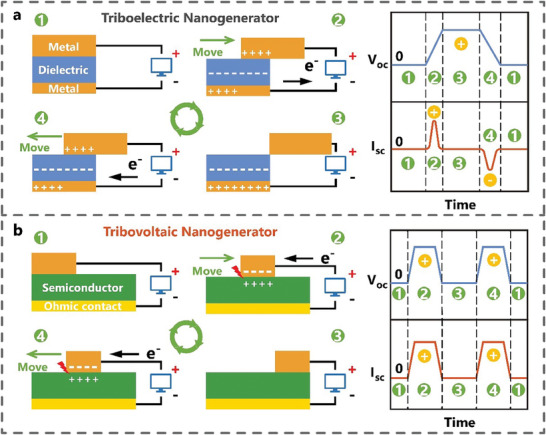
Differences of sliding‐mode tribovoltaic nanogenerator and triboelectric nanogenerator.

TENG utilizes metal electrodes and insulator materials, such as polymers, to generate alternating current based on the coupling effect of contact electrification and electrostatic induction (**Figure** **3**a). When the metal comes into contact with the dielectric material, due to the different electronegativity of the metal and the dielectric material (assuming that the metal tends to lose electrons more than the dielectric material, taking the sliding mode TENG as an example), the electrons on the surface of the metal transfers to the surface of the dielectric material. It causes the surface of the metal to become positively charged, while the surface of the dielectric material becomes negatively charged in equal amounts. In the initial state, with complete overlap between the metal and the dielectric material, no current flows in the external circuit due to the balance of the positive and negative charges. (Figure 3a‐step 1). When the upper metal layer slides to the right, the contact area between the metal and the dielectric material decreases. This reduction in contact area causes charges on the contact surfaces to separate, leading to electrostatic induction and the creation of a potential difference between the upper and lower metal layers. Consequently, electrons flow from the lower metal layer to the upper metal layer, resulting in the generation of a current in the external circuit with positive current direction (Figure 3a‐step 2). During the right sliding process, the charge continues to flow in the outer circuit, and the number of separated charges increases until the metal is completely separated from the dielectric material. At this point, the number of transfer charge between the upper and lower metal layers reaches its maximum (Figure 3a‐step 3). When the upper metal layer slides to the left, the contact area between the metal and the dielectric material increases. To maintain electrostatic equilibrium, electrons flow from the upper metal layer to the lower metal layer through the external circuit, generating a current with a negative current direction. During the left sliding process, the number of separated charges decreases, and the potential difference between the upper and lower metal layers gradually decreases until the metal and dielectric material are in complete contact again (Figure 3a‐step 4). Throughout the entire cycle, the sliding process is symmetrical, causing a pair of symmetrical alternating current peaks. In addition, the material/ion charge transfer is also an important reason for the electricity generation of the TENG.^[^ ^41^ ^]^

However, the TVNG based on dynamic semiconductor interfaces can generate DC output, which presents a different working principle from TENG (Figure 3b). In the initial state, the metal slider remains stationary and is in contact with the semiconductor under load pressure. Taking the sliding metal‐semiconductor interface for example, electrons with high Fermi energy levels flows from the semiconductor to the metal due to the Fermi energy difference between metal and semiconductor materials (assuming that the Fermi energy level of semiconductor is higher than that of the metal). It leads to a negative charge on the metal surface and a positive charge on the semiconductor surface and also causes an electric field at the metal‐semiconductor contact interface (Figure 3b‐step 1). When the metal slides to the right on the semiconductor surface, friction energy excites the non‐equilibrium carriers in the frictional interface. The electron‐hole pairs excited by the friction energy separate and move in the direction of the electric field. Electrons flow from the semiconductor to the metal, forming a positive current and voltage in the external circuit (Figure 3b‐step 2). When the metal stops at the end of the semiconductor, no carriers are excited by friction energy, resulting in zero current and voltage in the external circuit (Figure 3b‐step 3). When the metal begins to slide to the left, the electric field direction and the motion direction of the frictionally excited non‐equilibrium carriers remain unchanged. Electrons still flow from the semiconductor to the metal, creating a positive current and voltage in the external circuit (Figure 3b‐step 4). Therefore, the TVNG based on semiconductor materials can generate a DC signal during the sliding process. Compared with TENG, the typical output characteristics of TVNG are high current density (≈A m^−2^) and low impedance (≈kΩ). The specific comparison between TENG and TVNG is shown in **Table** **1**.

**Table 1:** Comparison of material, structure and property of TENG and TVNG.

	TENG	TVNG
Friction Material	Insulator	Semiconductor
Device Structure	Contact and Separation	In‐plane Friction
Mechanism	Contact Electrification	Friction Excited Carriers
Output Mode	Alternating Current	Direct Current
Output Characteristic	Low Current Density (nA‐µA/cm^2^)	High Current Density (mA‐A/cm^2^)
Matching Impedance	High Impedance (MΩ‐GΩ)	Low Impedance (Ω‐MΩ)

Here, we systematically summarize the current development of tribovoltaic effect based on semiconductor materials. The second section provides an overview of the research progress in TVNGs based on various interfaces, including metal‐semiconductor, metal‐insulator‐semiconductor, semiconductor‐semiconductor, liquid‐solid and flexible interface. In the third section, we summarize the output characteristics of TVNG and explore both internal and external factors that influence the output of TVNG. In the fourth section, we summarize the diverse mechanistic explanations of the tribovoltaic effect, focusing on the influence of built‐in electric fields and interfacial electric fields. In the fifth section, we discuss the coupling mechanism of tribovoltaic effect and multi‐physical field effect for multi‐source energy harvesting. In the sixth section, we highlight specific applications of tribovoltaic devices in various fields, showcasing their potential applications. In the seventh section, four important future research directions and challenges related to the tribovoltaic effect are pointed out.

## 2 Materials and Structures of Tribovoltaic Nanogenerator

### 2.1 Metal‐Semiconductor (MS) Interface

**Figure 4:**
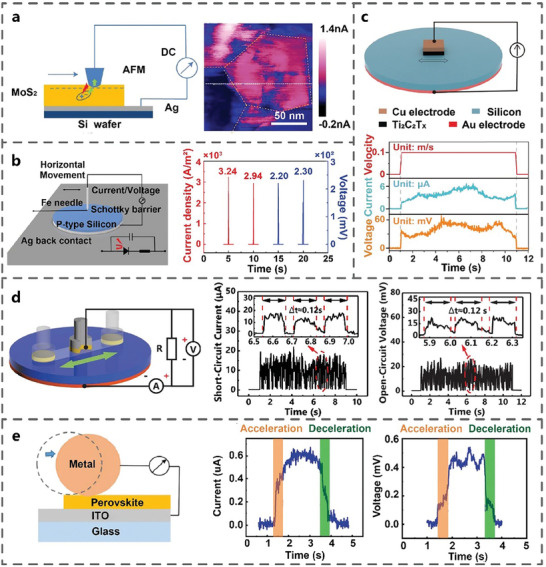
TVNGs based on metal‐semiconductor interface. a) Pt/Ir tips‐MoS_2_ films (Adapted from Liu et al.^[^ ^21^ ^]^ with permission. Copyright 2018, Nature Publishing Group). b) Fe‐Si (Adapted from Lin et al.^[^ ^42^ ^]^ with permission. Copyright 2019, WILEY‐VCH Verlag GmbH & Co. KGaA, Weinheim). c) MXene‐Si (Adapted from Luo et al.^[^ ^43^ ^]^ with permission. Copyright 2022, WILEY‐VCH Verlag GmbH & Co. KGaA, Weinheim). d) 304 stainless steel‐Si (Adapted from Zhang et al.^[^ ^27^ ^]^ with permission. Copyright 2020, WILEY‐VCH Verlag GmbH & Co. KGaA, Weinheim). e) Al‐perovskite (Adapted from Yuan et al.^[^ ^37^ ^]^ with permission. Copyright 2022, WILEY‐VCH Verlag GmbH & Co. KGaA, Weinheim) and their electric output performance.

The tribovoltaic effect was first discovered at the metal‐semiconductor interface. In 2018, Liu et al. first observed the DC output at the interface of sliding metal and MoS_2_ films by atomic force microscope (AFM) (**Figure** **4**a). A DC signal of 0.1–1 nA was found by a conductive AFM tip with a contact force of 5 nN and zero bias. The nanoscale contacts sliding Schottky junction could directly generate a maximum current density of 10^6^ A m^−2^.^[^ ^21^ ^]^ Later, similar phenomena were found in silicon‐based systems. Lin et al. designed a TVNG based on ferrum (Fe) tip and a smooth Si wafer, which reached a peak current density of 3.2 × 10^3^ A m^−2^ and a voltage of ≈230 V at the contact area of 0.1 mm^2^ (Figure 4b).^[^ ^42^ ^]^ Subsequent studies extended the nanoscale contact points to centimeter‐scale surface contacts. In 2022, Luo et al. extended the metal friction layer material to 2D metal carbide, creating a high performance TVNG composed of MXene film and Si. When the MXene film was rubbed on a Si wafer, it generated an open‐circuit voltage (V_oc_) up to 0.06 V and a short‐circuit current (I_sc_) up to 6 µA (Figure 4c).^[^ ^43^ ^]^ To investigate the tribovoltaic characteristics between metals and semiconductors, Zhang et al. used n‐type Si (nSi)‐stainless steel dynamic heterostructure to measure the output characteristics of TVNG under lateral sliding (Figure 4d). The average V_oc_ reached 10–20 mV, and the I_sc_ reached 10–20 µA.^[^ ^27^ ^]^ Subsequently, Yuan et al. designed a rolling TVNG based on aluminum (Al)‐CsPbBr_3_, where the Al roller rolled on the CsPbBr_3_ film in one direction through acceleration, constant velocity, and deceleration processes. It corresponded to the rising, leveling off, and falling processes of the output curve, and the variable speed process effectively excited electron‐hole pairs and promoted carrier transport (Figure 4e).^[^ ^37^ ^]^ It is evident that TVNG based on metal‐semiconductor dynamic interface is a general tribovoltaic phenomenon under different friction materials, contact scales, and motion modes.

### 2.2 Semiconductor–Semiconductor Interface

**Figure 5:**
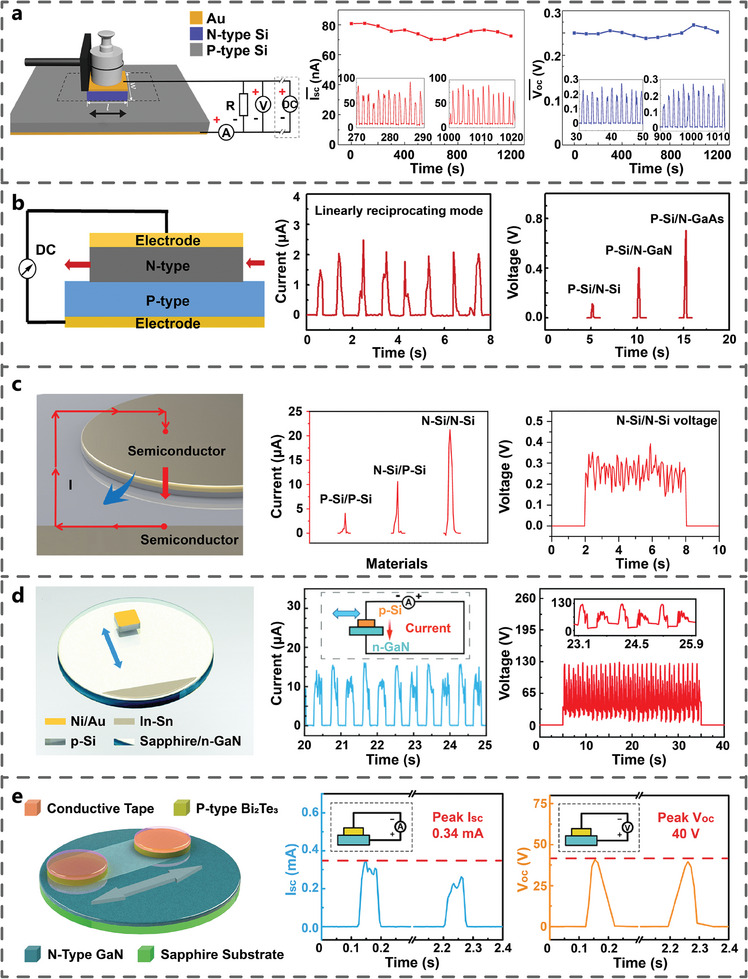
TVNGs based on semiconductor‐semiconductor interface. Electric output performance of a) nSi and pSi (Adapted from Xu et al.^[^ ^24^ ^]^ with permission. Copyright 2019). b) different n‐type semiconductor and p‐type semiconductor (Elsevier B.V. Adapted from Lu et al.^[^ ^44^ ^]^ with permission. Copyright 2019, Elsevier Inc). c) different nSi and pSi (Adapted under terms of the CC‐BY license.^[^ ^45^ ^]^ Copyright 2020, Lu et al., published by American Association for the Advancement of Science). d) nGaN and pSi (Adapted from Wang et al.^[^ ^35^ ^]^ with permission. Copyright 2022, The Royal Society of Chemistry). e) uGaN and pBi_2_Te_3_ (Adapted from Zhang et al.^[^ ^34^ ^]^ with permission. Copyright 2022, WILEY‐VCH Verlag GmbH & Co. KGaA, Weinheim).

With further investigation, the researchers discovered that the tribovoltaic effect also in the sliding interface between semiconductors. In 2019, Xu et al. found that the sliding interface of the nSi and pSi generated I_sc_ of ≈80 nA and V_oc_ of 0.25 V, with no significant output degradation observed (**Figure** **5**a). Current flowed from the pSi to the nSi via an external circuit, and once the sliding stopped, the current instantaneously dropped to zero.^[^ ^24^ ^]^ In the same year, Lu et al. designed a typical TVNG based on dynamic gallium arsenide (GaAs)‐Si, where a nGaAs wafer was pressed tightly onto a pSi substrate, generating a short‐circuit current density (J_sc_) of 1.5 mA cm^−2^ under the pressure of 6 N. This TVNG generated I_sc_ of ≈2 µA in reciprocating mode, which produced an V_oc_ of nearly 0.7 V (Figure 5b).^[^ ^44^ ^]^ In 2022, Lu et al. investigated the electric output characteristics of TVNG based on PP, PN and NN Si homojunctions under identical friction conditions. They obtained short‐circuit current of 4.1, 10.6, and 21.4 µA, respectively. Among these, the homojunction with NN configuration exhibited the highest current, attributed to the higher carrier mobility in the NN homojunction compared to the PN and PP homojunctions (Figure 5c).^[^ ^45^ ^]^ In 2022, Wang et al. used the interface based on pSi and n‐type gallium nitride (nGaN) to obtain TVNG, whose I_sc_ reached 15 µA during the rubbing process (Figure 5d). The continuous output of 130 V was achieved at a pressure of 8 N, accomplishing ultra‐high V_oc_ output of TVNG.^[^ ^35^ ^]^ In the same year, Zhang et al. proposed a TVNG based on p‐type Bi_2_Te_3_ and nGaN. When a pressure of 4 N was applied to the GaN slider and reciprocated at a maximum speed of 50 cm s^−1^ for 3 cm, it was observed that the peak V_oc_ was ≈40 V and the peak I_sc_ reached 0.34 mA (Figure 5e).^[^ ^34^ ^]^ The phenomenon of DC power generation based on the SS interface further confirms the tribovoltaic effect and realizes the output of ultrahigh voltage and power density, which is expected to become a new approach for friction dissipation energy harvesting.

### 2.3 Metal‐Insulator‐Semiconductor or Semiconductor‐Insulator‐Semiconductor Interface

**Figure 6:**
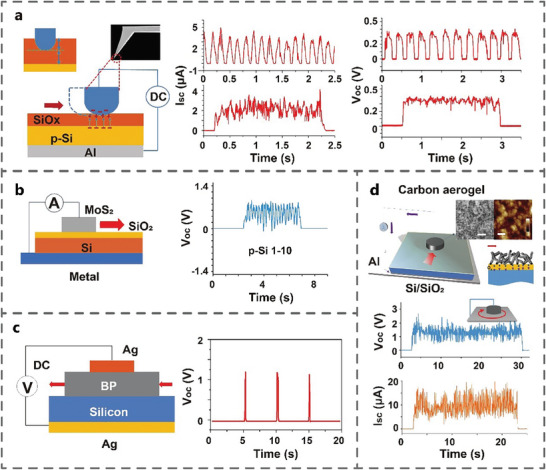
TVNGs based on metal‐insulator‐semiconductor (MIS) and semiconductor‐insulator‐semiconductor (SIS) interfaces. a) AFM tips‐SiO_x_‐p‐Si (Adapted from Liu et al.^[^ ^22^ ^]^ with permission. Copyright 2018, Elsevier B.V.). b) MoS_2_‐SiO_2_‐Si (Adapted from Liu et al.^[^ ^46^ ^]^ with permission. Copyright 2019, American Chemical Society). c) Ag‐black phosphorus‐Si (Adapted under terms of the CC‐BY license.^[^ ^25^ ^]^ Copyright 2019, Lu et al., published by American Association for the Advancement of Science). d) Carbon aerogel‐SiO_x_‐Si (Adapted from Zhang et al.^[^ ^47^ ^]^ with permission. Copyright 2019, WILEY‐VCH Verlag GmbH & Co. KGaA, Weinheim) and their electric output performance.

Subsequently, the researchers tried to add high‐impedance insulators into MS and SS interfaces, revealing that the interfaces based on metal‐insulator‐semiconductor (MIS) and semiconductor‐insulator‐semiconductor (SIS) also exhibit tribovoltaic effect. In 2018, Liu et al. found that the tribovoltaic effect existed at the sliding friction interface of MIS. A steel probe was used to slide on a pSi substrate with a naturally grown silicon dioxide (SiO_2_) insulator layer. The electrical output was tested with a linear reciprocal and circular rotation movement by a probe. The V_oc_ reached 300–400 mV and the I_sc_ reached 3–5 µA, with J_sc_ as high as 3–5 A m^−2^ (**Figure** **6**a).^[^ ^22^ ^]^ In 2019, Liu et al. investigated TVNG with MIS interface consisting of MoS_2_ films and Si sheets. During the rubbing process, the TVNG consist of the pSi with resistivity of 1–10 Ω cm produced nearly 0.8 V of V_oc_ (Figure 6b).^[^ ^46^ ^]^ In the same year, Lu et al. designed a TVNG based on dynamic black phosphorus‐insulator‐Si, where the black phosphorus moved on the Si substrate with a maximum V_oc_ of 1.2 V, peak I_sc_ of 6.2 µA, and J_sc_ of 124 A m^−2^ (Figure 6c).^[^ ^25^ ^]^ Then, Liu et al. used carbon aerogel‐Si to form multiple nano‐contacts, which increased the I_sc_ and V_oc_ of TVNG. Since the carbon aerogel was composed of nanoscale carbon matrix, the surface of the carbon aerogel was covered with nano‐protrusions. Therefore, the carbon aerogel naturally formed flexible nano‐contacts with the semiconductor substrate under rubbing. The V_oc_ up to 2 V and the I_sc_ up to 15 µA were measured under circular motion (Figure 6d).^[^ ^47^ ^]^ The MIS and SIS interfaces are created by adding insulation layer, which provides a new insights for studying the mechanism and characteristics of tribovoltaic effect.

### 2.4 Liquid‐Solid (LS) Interface

**Figure 7:**
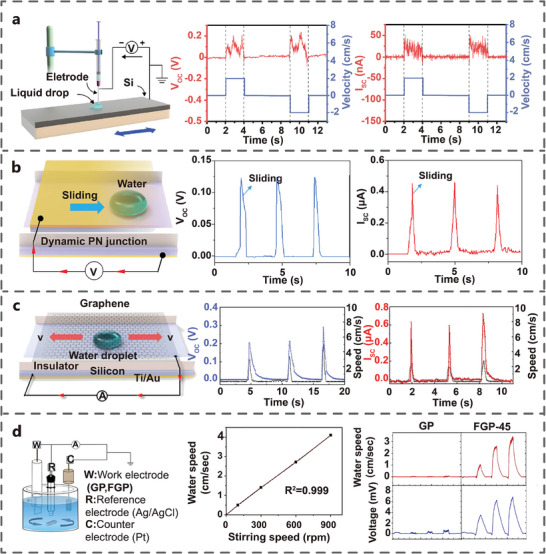
TVNGs based on liquid and semiconductor (LS) interface. a) DI water‐Si (Adapted from Yang et al.^[^ ^29^ ^]^ with permission. Copyright 2020, Elsevier B.V.). b) Si‐water‐Si (Adapted under terms of the CC‐BY license.^[^ ^48^ ^]^ Copyright 2021, Lu et al., published by American Association for the Advancement of Science). c) Graphene‐ polarized water‐Si (Adapted from Liu et al.^[^ ^49^ ^]^ with permission. Copyright 2021, American Chemical Society). d) Fluorinated graphite paper‐water (Adapted from Huang et al.^[^ ^50^ ^]^ with permission. Copyright 2022, Elsevier B.V.) and their electric output characteristics.

As research on the tribovoltaic effect advances, the exploration of friction interface has expanded from solid‐solid friction to liquid‐solid friction. In 2020, Lin et al. chose deionized (DI) water as the liquid friction layer to investigate the electric output of TVNG at water‐silicon interface. Controlled by syringe, DI droplet enabled relative sliding between DI water and the Si wafer (**Figure** **7**a). The V_oc_ was ≈200 mV, and the I_sc_ was ≈40 nA.^[^ ^29^ ^]^ In 2021, Lu et al. developed a TVNG based on a dynamic semiconductor‐water‐semiconductor interface, where the water droplet was positioned between nSi and pSi (Figure 7b). The water droplet moved back and forth in the channel formed by the nSi and pSi, which generated V_oc_ of 0.12 V and I_sc_ of 0.46 µA with a volume of 50 µL sliding at a speed of 150 mm s^−1^.^[^ ^48^ ^]^ Later, Yan et al. designed the gap interface of graphene film and nSi. A DC output was generated from the polarized water‐graphene‐Si interface (Figure 7c). The V_oc_ and I_sc_ correlated with droplet velocity change as the droplet moved and reached the maximum values of ≈0.3 V and ≈0.7 µA.^[^ ^49^ ^]^ In 2022, Huang et al. designed a new immersed TVNG and investigated the output performance of graphite paper (GP) and fluorinated graphite paper (FGP) under the water (Figure 7d). The FGP demonstrated significantly higher electric output than that of GP. At the water velocity of 1.2 cm s^−1^, the J_sc_ and V_oc_ of the FGP‐water TVNG were 3.3 µA cm^−2^ and 65 mV.^[^ ^50^ ^]^ The study of TVNG based on LS interface enriches the understanding of LS contact electrification mechanisms, providing a new solution for raindrop energy harvest.

### 2.5 Flexible Interface

**Figure 8:**
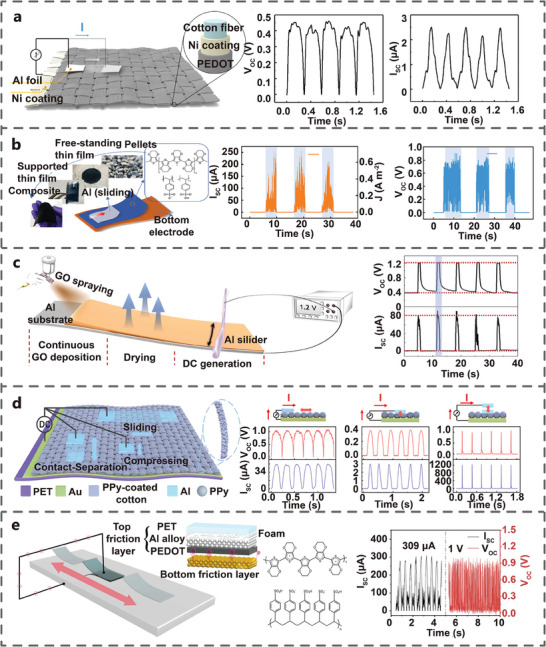
TVNGs based on conductive polymer. a) PEDOT:PSS with Ni‐coated textile electrode (Adapted from Meng et al.^[^ ^31^ ^]^ with permission. Copyright 2021, American Chemical Society). b) PEDOT:PSS with Fe electrode (Adapted from Yang et al.^[^ ^51^ ^]^ with permission. Copyright 2021, WILEY‐VCH Verlag GmbH & Co. KGaA, Weinheim). c) Al‐GO (Adapted from Chen et al.^[^ ^52^ ^]^ with permission. Copyright 2021, Elsevier B.V.). d) Al‐PPy (Adapted from Meng et al.^[^ ^53^ ^]^ with permission. Copyright 2022, The Royal Society of Chemistry). e) PEDOT:PSS with Al electrode (Adapted from You et al.^[^ ^54^ ^]^ with permission. Copyright 2021, Elsevier B.V.) and their electric output performance.

Due to the inherent rigid properties of inorganic semiconductor, their application in the field of wearable electronics is constrained. Therefore, a growing number of researchers have shifted their focus toward the exploration of flexible TVNG based on organic semiconductor. In 2021, Meng et al. designed a TVNG with dynamic metal‐polymer semiconductor interface using metallic Al and lightly doped Poly (3,4‐ethylene dioxythiophene): poly (styrene sulfonate) (PEDOT: PSS). When a thin Al foil slid over the PEDOT: PSS coated fabric, the external circuit generated a current from the Nickel (Ni) coating to the Al foil. The V_oc_ and I_sc_ amplitudes reached 0.45 V and 2.5 µA, respectively. (**Figure** **8**a).^[^ ^31^ ^]^ In the same year, Yang et al. investigated the electrical output characteristics of a TVNG in rotational sliding mode using Al foil and PEDOT:PSS film. The V_oc_ reached 0.8 V and the I_sc_ reached 200 µA, corresponding to J_sc_ of 0.73 A m^−2^ (Figure 8b).^[^ ^51^ ^]^ Later, Chen et al. prepared flexible TVNG utilizing Al wire with asymmetric graphene oxide (aGO). The DC output was detected when the Al slider slid continuously on aGO film. At the relative humidity (RH) of 70% and sliding speed of 60 mm s^−1^, the V_oc_ increased from 0.4 to 1.2 V and the I_sc_ increased from 0 µA to 80 µA (Figure 8c).^[^ ^52^ ^]^ In 2022, Meng et al. prepared contact‐separation mode TVNG based on a new organic semiconductor polymer polypyrrole (PPy). In the sliding motion mode, the peak V_oc_ was ≈1.06 V, and the I_sc_ was 51.52 µA. In the compressive motion mode, the V_oc_ could reach 0.36 V, and I_sc_ was 3.03 µA. When the Al film is subjected to vertical contact separation motion on the PPy fabric, the V_oc_ and I_sc_ reached 1.06 V and 1.26 mA, respectively, with the J_sc_ reaching as high as 16 A m^−2^ (Figure 8d).^[^ ^53^ ^]^ In the same year, You et al. studied the effect of various metal friction layer materials on the tribovoltaic effect using PEDOT:PSS as an organic semiconductor layer. The TVNG based on PEDOT: PSS top layer and Al bottom layer exhibited the best performance under the same measurement conditions. The optimal V_oc_ and I_sc_ of the TVNG were 1 V and 309 µA at a sliding speed of 30 cm s^−1^ and a load pressure of 30 N (Figure 8e).^[^ ^54^ ^]^ The research of flexible semiconductor materials has broadened the application of TVNG in the wearable field, providing an effective strategy for harvesting mechanical energy and implementing self‐powered electronics.

## 3 Characteristics of Tribovoltaic Nanogenerator

### 3.1 Electric Output Characteristics

**Figure 9:**
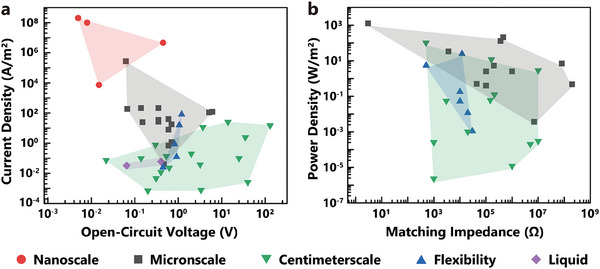
Typical electrical output characteristics of different types of TVNG. a) Short‐circuit current density versus open‐circuit voltage. b) Peak power density versus matching impedance. (Above data comes from Table 2).

At present, the TVNG can be divided into five types according to the size of effective contact area and the types of material: nanoscale (AFM tips), microscale (needle tips), centimeterscale (block sliders), flexibility (conducting polymers), liquid (water). The corresponding values for V_oc_, J_sc_, matching impedance, maximum power and contact area are shown in **Table** **2**. It is found that the contact scale has an important influence on the electrical output characteristics of TVNG (**Figure** **9**a). Nanoscale TVNG exhibits the highest J_sc_ (2 × 10^8^ A m^−2^) due to its larger electric field and better contact. The J_sc_ of microscale TVNG is between that of nanoscale and centimeterscale TVNG. Although the J_sc_ of centimeterscale TVNG is the smallest among several TVNGs, the highest J_sc_ can still reach 25 A m^−2^. Additionally, centimeterscale TVNG typically produces higher V_oc_, which is up to ≈140 V. Flexible TVNG is typically based on organic materials and has a higher J_sc_ than the TVNG made of inorganic materials. The liquid TVNG generally exhibits lower J_sc_ and V_oc_, due to the smaller contact force. In general, the power density of micron TVNG is higher than that of centimeterscale TVNG (Figure 9b). The matching impedance of the centimeterscale TVNG varies from 10^3^ to 10^7^ Ω, with the highest power density reaching up to 102.4 W m^−2^. The matched impedance and maximum power density of various centimeterscale TVNGs differ significantly due to the difference in friction pair materials. In microscale TVNG, most of the impedance typically ranges from 10^5^ to 10^6^, and the lowest impedance can reach several Ω. The maximum power density is generated by microscale TVNG (2000 W m^−2^). The matching impedance of flexibility TVNGs is ≈10^3^–10^4^ Ω, with the power density not exceeding 50 W m^−2^.

**Table 2:** Performance parameters of different types of TVNG.

Type	V_oc_(V)	I_sc_(µA)	P_m_ (µW)	Impedance(Ω)	Area(mm^2^)	Reference
Nano	0.0080	0.0006			6.00E‐12	[21]
Nano	0.0150	0.0002			3.15E‐08	[68]
Nano		0.0020				[55]
Nano		0.0306			1.06E‐07	[78]
Nano	0.0050	0.0060			3.00E‐11	[30]
Nano		0.0003				[56]
Nano	0.45	0.0002			4.60E‐05	[79]
Nano		0.00018				[80]
Centi	3.69	5.73			0.5	[37]
Centi	0.2000	0.0700			1.00E+02	[26]
Centi	2.0000	15.700	4.7600	146 000	7.85E+01	[47]
Centi	0.3100	0.4600	0.0012	1 000 000	1.00E+02	[24]
Centi	0.0220	15.000	0.0508	1000	2.00E+02	[27]
Centi	0.0020	1.4000	0.0005	1000	2.00E+02	[28]
Centi	0.4000	1.2000			1.00E+02	[81]
Centi	0.4500	2.5000	0.11	30 000	1.00E+02	[31]
Centi	35	320	1580	160 000	1.33E+02	[34]
Centi	0.83	216	13.42	10 000	2.54E+02	[51]
Centi	0.92	62	5.84	20 000	5.00E+02	[54]
Centi	3.35	0.154	0.06	10 000 000	2.00E+02	[82]
Centi	0.53	13.65			100	[57]
Centi	0.14	22			225	[43]
Centi	14	58	204.8	500	2	[83]
Centi	40	0.34	1576.05	160 000	133	[34]
Centi	130	15	280	10 000 000	100	[35]
Centi	25	10	0.02	5 000 000	100	[65]
Centi	3.2	15.06	12.32	210 000	100	[84]
Centi	1.06	1260	425.25	510	78.75	[53]
Centi	0.3	155	10.8	3000	200	[36]
Centi	0.62	9.2	0.4	20 000	400	[85]
Centi	0.8	11		10 000		[86]
Micro	0.6	0.016	0.00799	200 000 000	0.017	[61]
Micro	0.47	0.032	0.00296	7 000 000	0.8	[63]
Micro	0.139	0.0034	0.0001	8.00E+07	1.60E‐05	[32]
Micro	6.1000	6.2000	10.500	450 000	5.00E‐02	[25]
Micro	5.1000	5.6000	6.5000	360 000	5.00E‐02	[44]
Micro	0.3500	21.400	3.3600	3600	1.00E‐01	[87]
Micro	1.21 (77 K)	11.38 (77 K)				[33]
Micro	1.2	81.06	24.08	12 000	1.00E+00	[52]
Liquid	0.4000	0.3000			5.00E+00	[29]
Liquid	0.2700	0.3000				[69]
Liquid	0.1120	0.4400	0.0229	390 000		[48]
Liquid	0.3000	0.8000	0.0870	250 000		[49]
Liquid	0.15	0.07				[60]
Liquid	0.065	1.2				[50]
Liquid		0.03				[88]
Needle	0.3500	2.7000	0.2500	100 000	1.00E‐01	[22]
Needle	0.6000	4.0000	0.5250	200 000	1.00E‐01	[58]
Needle	0.3500	0.0687	0.0049	1 000 000	1.96E‐03	[89]
Needle	0.7000	0.5500			3.00E‐02	[67]
Needle	0.1500	0.7854	0.0157	44 000	3.14E‐02	[26]
Needle	0.7000	2.0000			1.67E+00	[46]
Needle	0.0620	27 560	126.20	3	1.00E‐01	[42]
Needle	0.6000	0.8000	0.0400	100 000	1.00E‐01	[90]
Needle	0.0671	7600			4.00E+01	[59]
Needle		0.0032	0.0050		2.50E‐06	[56]
Needle		0.0487			0.0017393	[71]
Needle	0.25	1.41				[38]

### 3.2 Influences of Materials

#### 3.2.1 Solid and Solid Type

**Figure 10:**
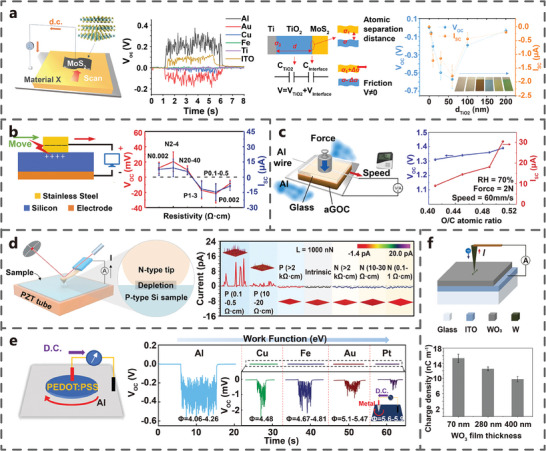
The influence of material factors on the electrical output of solid‐solid TVNG. a) Metal type and TiO_2_ thickness (MoS_2_‐metal) (Adapted from Liu et al.^[^ ^46^ ^]^ with permission. Copyright 2019, American Chemical Society). b) P/N type and resistivity of Si (metal‐Si) (Adapted from Zhang et al.^[^ ^27^ ^]^ with permission. Copyright 2020, WILEY‐VCH Verlag GmbH & Co. KGaA, Weinheim). c) The ratio of oxygen atom to carbon atom (aGOC‐Al) (Adapted from Chen et al.^[^ ^52^ ^]^ with permission. Copyright 2021, Elsevier B.V.). d) P/N type and resistivity of Si (AFM probe‐Si) (Adapted from Zhang et al.^[^ ^55^ ^]^ with permission. Copyright 2020, WILEY‐VCH Verlag GmbH & Co. KGaA, Weinheim). e) Work functions (metal‐conductive polymer) (Adapted from Yang et al.^[^ ^51^ ^]^ with permission. Copyright 2021, WILEY‐VCH Verlag GmbH & Co. KGaA, Weinheim). f) WO_3_ thickness (WO_3_‐tungsten probes) (Adapted from Šutka, A. et al.^[^ ^56^ ^]^ with permission. Copyright 2021, American Chemical Society).

Thickness of dielectric layer: Liu et al. investigated the effect of the thickness of the insulating layer (TiO_2_) on the MoS_2_‐Ti TVNG (**Figure** **10**a). When the thickness of TiO_2_ varied from 5 to 200 nm, both the V_oc_ and I_sc_ of TVNG initially increased to maximum and then gradually decreased. When the thickness of TiO_2_ is 60 nm, the V_oc_ and I_sc_ both reach the maximum of ≈0.7 V and ≈2 µA, respectively. The surface potential between TiO_2_ and Ti is significantly different with the TiO_2_ thickness increasing, contributing to an increment in V_oc_ and I_sc_. Further increasing the thickness of TiO_2_ resulted in a decrease in charge difference in the dual capacitance model and an increase in the internal resistance of TVNG, which led to a decrease in V_oc_ and I_sc_.^[^ ^46^ ^]^

Resistivity of semiconductors: Zhang et al. investigated the electrical output characteristics of Si‐stainless‐steel TVNG by altering the resistivity and doping type of Si (Figure 10b). They observed distinct directions in V_oc_ and I_sc_ for P‐type Si and N‐type Si. Regardless of P‐type Si or N‐type Si, both V_oc_ and I_sc_ increased first and then decreased with the rise of resistivity, which is attributed to the difference of work function between Si and stainless steel.^[^ ^27^ ^]^ Zheng et al. studied the influence of the P/N type and resistivity on the V_oc_ and I_sc_ (Figure 10c). They noted that the tip‐nSi TVNG struggled to generate current due to the weak built‐in electric field of NN heterojunction. The higher the resistivity of Si led to a sharp decrease in carrier numbers at room temperature, resulting in a low current.^[^ ^55^ ^]^

Ratio of organic semiconductors: Chen et al. studied the influence of the atomic ratio on the aGO‐Al TVNG. It is found that the V_oc_ and I_sc_ of the TVNG gradually increases with the increase of O atom ratio (Figure 10d). This increase is attributed to the change in the atomic ratio of O and C, which affects the number of cationic functional groups.^[^ ^52^ ^]^

Work function of metal: Liu et al. discovered that metal‐MoS_2_ TVNG had different V_oc_ values and directions (Figure 10a).^[^ ^46^ ^]^ Meanwhile, they found that the V_oc_ and I_sc_ of metals‐ PEDOT: PSS TVNG are also different (Figure 10e). This discrepancy is attributed to the difference of work function between the two materials, impacting the size of the built‐in electric field of TVNG.^[^ ^51^ ^]^

Thickness of semiconductors: Andris et al. studied the effect of different WO_3_ thickness on the V_oc_ and I_sc_ of WO_3_‐W TVNG (Figure 10f). They found a decrease in the V_oc_ and I_sc_ of TVNG with the increasing of WO_3_ thickness. The phenomenon can be attributed to the fact that a thinner film provides a shorter path for carrier transfer, reducing the possibility of their scattering.^[^ ^56^ ^]^

#### 3.2.2 Solid and Liquid Type

**Figure 11:**
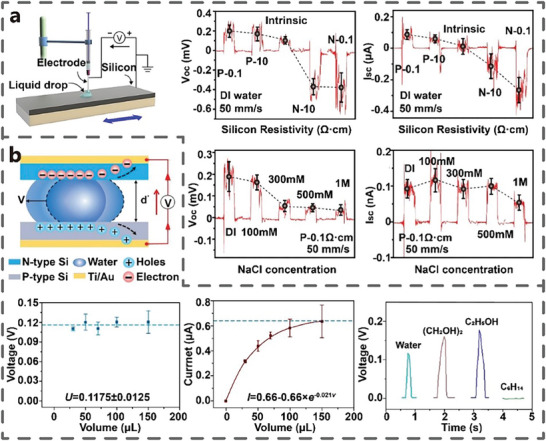
The influence of material factors on the V_oc_ and I_sc_ of liquid‐solid TVNG. a) Resistivity of Si and NaCl solution concentration (liquid‐Si) (Adapted from Lin et al.^[^ ^29^ ^]^ 100 with permission. Copyright 2020, Elsevier B.V.). b) Droplet size and liquid type (metal‐Si TVNG) (Adapted under terms of the CC‐BY license.^[^ ^48^ ^]^ Copyright 2021, Lu et al., published by American Association for the Advancement of Science).

Resistivity of semiconductor: Lin et al. found that the differences in P/N type and resistivity of Si can generate different magnitude and directions of V_oc_ and I_sc_ (**Figure** **11**a), which is consistent with the direction of built‐in electric field. Changes in the PN type and resistivity of Si lead to alterations in the difference of work function between Si and deionized water.^[^ ^29^ ^]^

Concentration of solution: Lin et al. also found that the V_oc_ of TVNG decreased with the increase of the concentration of NaCl solution (Figure 11a). With the increase of NaCl concentration, the electron transfer between NaCl solution and different media decreases, leading to a reduction in the number of excited electron hole pairs. However, the I_sc_ does not decrease significantly with the increase of solution concentration, owing to the enhanced conductivity of the solution.^[^ ^29^ ^]^

Droplet size: As the volume of deionized water gradually increases, the I_sc_ of TVNG gradually increases, while the V_oc_ remains almost unchanged (Figure 11b). The increase in the volume of deionized water results in more water molecules becoming polarized. However, V_oc_ does not increase further, owing to the constant Fermi energy difference between the two semiconductors.^[^ ^48^ ^]^

Liquid type: Lu et al. also observed that the V_oc_ of pSi‐water‐nSi TVNG, pSi‐glycol‐nSi TVNG, and pSi‐ethanol‐nSi TVNG is 0.12, 0.16, and 0.18 V, respectively, while pSi‐n‐hexane‐nSi TVNG had a V_oc_ of 0 V (Figure 11b). This observation is attributed to the fact that polar liquids (water, ethylene glycol, and ethanol) are more easily polarized, while non‐polar liquids (n‐hexane) are less easily polarized. They believed that TVNG made with polar liquids can produce higher V_oc_.^[^ ^48^ ^]^

### 3.3 Influences of Working Conditions

#### 3.3.1 Operating Factors

**Figure 12:**
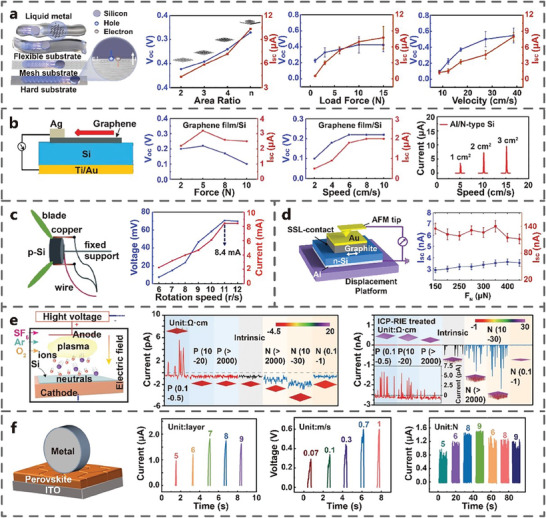
The influence of operating factors on the electrical output. a) Area, pressure and velocity (carbon aerogel‐Si) (Adapted from Xu et al.^[^ ^57^ ^]^ with permission. Copyright 2022, Elsevier B.V.). b) Area, pressure and velocity (graphite‐Si) (Adapted from Lin et al.^[^ ^58^ ^]^ with permission. Copyright 2019, WILEY‐VCH Verlag GmbH & Co. KGaA, Weinheim). c) Rotating speed (Cu‐Si) (Adapted under terms of the CC‐BY license.^[^ ^59^ ^]^ Copyright 2021, Yu et al., published by Royal Society of Chemistry). d) Pressure in the superlubrication state (graphite‐Si) (Adapted under terms of the CC‐BY license.^[^ ^32^ ^]^ Copyright 2021, Huang et al., published by Spring Nature). e) ICP treatment (AFM probe‐Si) (Adapted from Zhang et al.^[^ ^55^ ^]^ with permission. Copyright 2020, WILEY‐VCH Verlag GmbH & Co. KGaA, Weinheim). f) Material thickness, velocity and pressure (rolling metal‐perovskite) (Adapted from Yuan et al.^[^ ^37^ ^]^ with permission. Copyright 2022, WILEY‐VCH Verlag GmbH & Co. KGaA, Weinheim).

Area: Xu et al. found that the contact area of Si‐Al TVNG was positively correlated with V_oc_ and I_sc_ (**Figure** **12**a).^[^ ^57^ ^]^ Similarly, Lin et al. found that the V_oc_ and I_sc_ of graphene‐silicon TVNG were also positively correlated with the contact area (Figure 12b).^[^ ^58^ ^]^

Velocity: When the speed increased from 8.66 to 38.73 cm s^−1^, the V_oc_ of Si‐Al TVNG gradually increased and then became saturated, while the I_sc_ increased linearly (Figure 12a).^[^ ^57^ ^]^ This phenomenon has also been found in the studies of other scholars.^[^ ^24^, ^28^, ^34^ ^]^ The I_sc_ and V_oc_ of Si‐graphene TVNG were positively correlated with the sliding speed (Figure 12b). With the increase of sliding speed, I_sc_ and V_oc_ first increase and then tend to saturation.^[^ ^58^ ^]^ The electrical output of the rotating mode TVNG developed by Yu et al. is also positively correlated with speed (Figure 12c). When the speed increases from 57 to 105 rpm s^−1^, the V_oc_ and I_sc_ of TVNG gradually increase from 7.2 mV and 2.3 mA to 70.7 mV and 8.4 mA, respectively. However, as the speed continues to increase, the V_oc_ and I_sc_ of TVNG remain stable.^[^ ^59^ ^]^ Yuan et al. investigated the effect of the rolling speed of Al on the electrical output of rolling mode TVNG on perovskite (Figure 12e). They found that the the V_oc_ and I_sc_ of TVNG gradually increases and tends to saturation with the increase of Al rolling speed. Due to the increase in sliding/rotating/rolling speeds, the friction interface will excite more charge carriers, resulting in a rise in electrical output. However, when the speed continues to increase, the electrical output will reach saturation due to the loss of friction material mass.^[^ ^37^ ^]^

Normal force: The normal force is positively correlated with its electrical output in Al‐Si TVNG (Figure 12a). With the increase of the force, more friction energy can be generated, and more carriers can be excited, increasing the electrical output of TVNG.^[^ ^57^ ^]^ However, Lin et al. found that when the normal force increases from 2 to 10 N, the I_sc_ and V_oc_ of TVNG first increase and then decrease (Figure 12b). As the normal force further increasing, the graphene film will be worn, which affects the magnitude of the electrical output.^[^ ^58^ ^]^ Huang et al. studied the influence of pressure on the electrical output of TVNG under the superlubricity state (Figure 12d). They found that the I_sc_ of TVNG gradually increased with the increasing normal force, but the V_oc_ remained almost unchanged.^[^ ^32^ ^]^ Similarly, the magnitude of normal force also affects V_oc_ and I_sc_ of TVNG in the rolling mode (Figure 12e). With the increase of normal force, the electrical output increases gradually, because the contact between Al and perovskite becomes closer and closer. However, when the pressure is greater than 3N, the V_oc_ and I_sc_ gradually decreases due to the destruction of the perovskite surface.^[^ ^37^ ^]^

Surface treatment: Zheng et al. found that the electrical output of the Si‐Pt TVNG after inductively coupled plasma treatment is higher than that of the untreated Si‐Pt TVNG (Figure 12f). The I_sc_ of surface treated TVNG can reach up to 2000 pA, which is several orders of magnitude higher output than that of untreated TVNG. This enhancement is attributed to the higher surface state density of treated TVNG.^[^ ^55^ ^]^

#### 3.3.2 Environmental Factors

**Figure 13:**
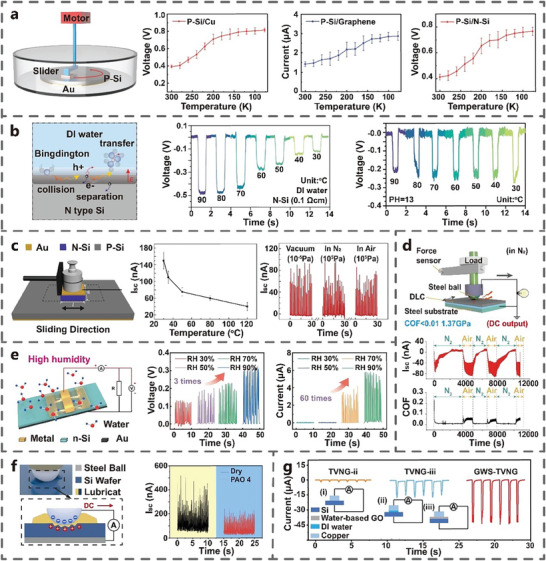
The influence of environmental factors on the electrical output. a) Low temperature (Si‐Cu/graphene/Si) (Adapted from Zheng et al.^[^ ^33^ ^]^ with permission. Copyright 2021, WILEY‐VCH Verlag GmbH & Co. KGaA, Weinheim). b) Temperature and PH (liquid‐Si) (Adapted from Zheng et al.^[^ ^60^ ^]^ with permission. Copyright 2021, WILEY‐VCH Verlag GmbH & Co. KGaA, Weinheim). c) Temperature, vacuum degree and gas environment (Si‐Si) (Adapted from Xu et al.^[^ ^24^ ^]^ with permission. Copyright 2019, Elsevier B.V.). d) Gas environment under superlubricity state (steel‐DLC) (Adapted from Zhang et al.^[^ ^61^ ^]^ with permission. Copyright 2022, Elsevier Inc). e) Humidity (Cu‐Si) (Adapted under terms of the CC‐BY license.^[^ ^62^ ^]^ Copyright 2022, Wang et al., published by Royal Society of Chemistry). f) Lubricating oil (steel‐Si) (Adapted from Yang et al.^[^ ^63^ ^]^ with permission. Copyright 2023, Elsevier B.V.). g) The type of lubricating oil (Cu and Si) (Adapted from Qiao et al.^[^ ^36^ ^]^ with permission. Copyright 2022, WILEY‐VCH Verlag GmbH & Co. KGaA, Weinheim).

**Temperature**: Zheng et al. found that when the temperature decreased from 300 to 77 K, the electrical output of pSi/Cu, pSi/Graphene and pSi/nSi TVNG gradually increased and stabilized (**Figure** **13**a). The decrease in temperature led to an increase in carriers mobility, attributed to reduced lattice scattering and ionization doping scattering.^[^ ^33^ ^]^ Similarly, in the case of TVNG in solid‐liquid contact, temperature will also affect its electrical output. Zheng et al. found that the V_oc_ and I_sc_ of TVNG (liquid and Si) decreased when the temperature of deionized water decreased from 90 to 30 °C (Figure 13b).^[^ ^60^ ^]^ The I_sc_ of nSi‐pSi TVNG is negatively correlated with temperature (Figure 13c). As the temperature rises, the Fermi energy level of the two semiconductors will move toward the center of the band gap, resulting in the reduction of the built‐in electric field.^[^ ^24^ ^]^

**PH of liquid**: Zheng et al. also found that at high PH value, the V_oc_ of TVNG hardly changed with the decrease of temperature, but the I_sc_ still gradually decreased (Figure 13b). When the pH value of the solution is high, the redox reaction between NaOH and Si is strong, which results in the excitation of a large number of charge carriers at low temperatures.^[^ ^60^ ^]^

**Vacuum and gas environment**: Xu et al. found that vacuum and gas environment have little impact on the electrical output of TVNG (Figure 13c).^[^ ^24^ ^]^ However, Zhang et al. found that in the superlubricity state, the environmental changes involving N_2_ and Air can affect the friction coefficient, which can affect the electrical output of TVNG (Figure 13d).^[^ ^61^ ^]^

**Interface lubricant**: Wang et al. found that with increasing humidity, the V_oc_ and I_sc_ of nSi‐Cu TVNG increased by 3 and 60 times, respectively (Figure 13e).^[^ ^62^ ^]^ Yang et al. found that adding lubricating oil PAO4 to Si‐steel TVNG can generate a decrease in electrical output (Figure 13f).^[^ ^63^ ^]^ However, adding deionized water and water‐based graphene at the friction interface can increase the I_sc_ of pSi‐Cu TVNG. Especially, the addition of water‐based graphene increases the I_sc_ of TVNG by an order of magnitude and significantly improves its durability (Figure 13g).^[^ ^36^ ^]^

## 4 Mechanism Discussions

Friction at semiconductor interfaces involves multiple physical mechanisms. The tribovoltaic effect may be coupled with interface triboelectrification, atomic bonding, phonon excitation and other processes, so the power generation mechanism is complicated. In the past two years, researchers globally have given proposed various mechanism analyses on the tribovoltaic effect of DC power generation. There are two main viewpoints: the theory driven by the internal electric field and the mechanism dominated by the interface electric field.

### 4.1 Built‐In Electric Field Dominated Theory

**Figure 14:**
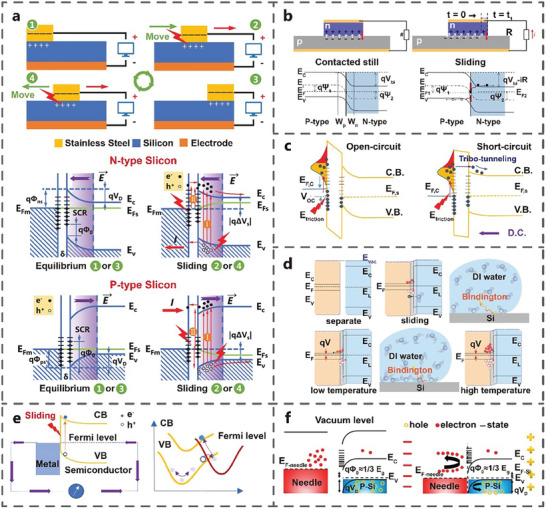
Mechanism analysis of TVNG. a) Atomic bonding and built‐in electric field (Adapted from Zhang et al.^[^ ^27^ ^]^ with permission. Copyright 2020, WILEY‐VCH Verlag GmbH & Co. KGaA, Weinheim). b) The built‐in electric field (Adapted from Xu et al.^[^ ^24^ ^]^ with permission. Copyright 2019, Elsevier B.V.). c) Electron tunneling (Adapted from Liu et al.^[^ ^47^ ^]^ with permission. Copyright 2019, WILEY‐VCH Verlag GmbH & Co. KGaA, Weinheim). d) “Bindington” (Adapted from Zheng et al.^[^ ^60^ ^]^ with permission. Copyright 2021, WILEY‐VCH Verlag GmbH & Co. KGaA, Weinheim). e) Ultra high local temperature (Adapted from Liu et al.^[^ ^64^ ^]^ with permission. Copyright 2022, Elsevier B.V.). f) Bounce between atomic electric field and built‐in electric field (Adapted under terms of the CC‐BY license.^[^ ^42^ ^]^ Copyright 2019, Lin et al., published by John Wiley and Sons).

Zhang et al. found that the electrical output of stainless steel‐Si TVNGs are different due to differences in the work function between the friction pairs. (**Figure** **14**a).^[^ ^27^ ^]^ They believe that the electrical output of the tribovoltaic effect is dominated by the built‐in electric field which is caused by the difference of the work function between stainless steel and Si. At first, the two electrodes remain relatively static. The electrons with higher Fermi energy level will flow from the semiconductor to the metal side, due to the Fermi energy level difference between the two materials (Figure 14a‐step 1). This made the metal surface negatively charged and the semiconductor surface positively charged, which generated a built‐in electric field. The formation and destruction of the atomic bond excites the electron‐hole pairs during the rubbing process. The electron‐hole pairs move directionally driven by the built‐in electric field, which generates current in the external circuit (Figure 14a‐step 2). When the metal slider stops sliding, no new carriers will be excited (Figure 14a‐step 3). The power generation process in Step 4 is similar to that in Step 2. This phenomenon exists not only in the dynamic Schottky contact, but also in the dynamic PN contact. Zhang et al. proposed in the nSi‐pSi TVNG that the built‐in electric field of PN heterojunction provides driving force for the directional movement of charge carriers (Figure 14b).^[^ ^24^ ^]^ Many scholars have extended on the basis of built‐in electric field, in TVNG with dielectric layer. Liu et al. propose the mechanism of tribovoltaic effect by carbon aerogel and Si covered with 1–2 nm SiO_2_ (Figure 14c).^[^ ^47^ ^]^ They believe that the friction excitation of non‐equilibrium carriers leads to the energy band shift in the Si depletion region in the open‐circuit condition. In the short‐circuit condition, the electrons may be transferred to the tunnel current in a non‐adiabatic way. Electrons can then pass through the SiO_2_ layer and enter Si. The tribovoltaic effect of solid‐liquid interface also accords with the theory of built‐in electric field. Zheng et al. believed that the release of “bindington” excited the carrier in the water‐nSi TVNG (Figure 14d).^[^ ^60^ ^]^ Liu et al. found that the local high temperature caused by interface friction can generate electron‐hole pairs through electron‐phonon coupling providing a theoretical framework understanding the tribovoltaic effect from the perspective of quantum mechanics (Figure 14e).^[^ ^64^ ^]^ Lin et al. thought that the Schottky junction formed at the metal and Si contacted. The Schottky junction would be destroyed with sliding and the diffusion electrons cannot traverse the depletion layer leading to electrons and holes bouncing back from the charged surface state caused by dangling bonds or vacancies, resulting in DC (Figure 14f).^[^ ^42^ ^]^

### 4.2 Interfacial Electric Field Dominated Theory

**Figure 15:**
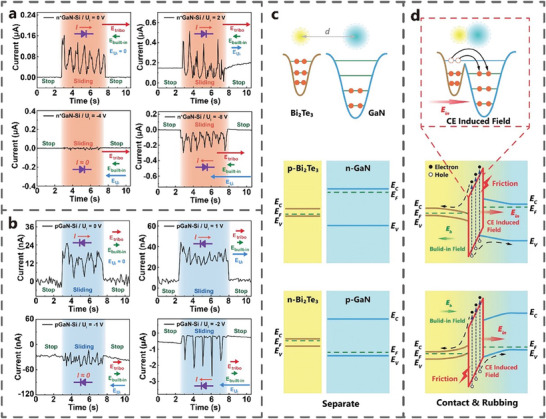
The leading role of interface electric field on carrier transport. The influence of the applied bias voltage on the a) nGaN‐Si TVNG and b) pGaN‐Si TVNG (Adapted from Chen et al.^[^ ^65^ ^]^ with permission. Copyright 2022, American Chemical Society). Electron cloud potential well model and energy band diagram in the state of c) separation and d) rubbing (Adapted from Zhang et al.^[^ ^34^ ^]^ with permission. Copyright 2022, WILEY‐VCH Verlag GmbH & Co. KGaA, Weinheim).

The theory of built‐in electric field has good applicability in Si‐based and narrow‐band semiconductors. In 2022, the tribovoltaic effect found in GaN‐Si TVNG is difficult to explain with the built‐in electric field theory. The direction of V_oc_ is opposite to the direction of the built‐in electric field, and its magnitude exceeded the band gap width. Therefore, Chen et al. proposed a new carrier transport theory.^[^ ^65^ ^]^ They postulated the existence of interfacial electric field from Si to n‐GaN, which is verified by the experiments involving applied bias voltage (**Figure** **15**a). At 0 V bias voltage, the current direction aligned with the interface electric field, but opposite the built‐in electric field. When the bias voltage increases to 2 V, the total electric field at the interface further intensified as the applied and the interfacial electric field aligned. The current is generated by applied electric field when the slider is stationary. During sliding, the current is the result of the combined action of applied electric field, built‐in electric field, and interface electric field. However, the minimum current during sliding is less than that at stop, due to the unstable contact of the two electrodes. Similarly, when a voltage opposite to the interfacial electric field is applied to the interface, the electrical output of TVNG will be suppressed. When the bias voltage is 4 V, the three electric fields cancel each other, and the total electric field intensity and the electrical output is ≈0. When the reverse bias voltage reaches 8 V, no current is generated in stationary, on account of the cut‐off state of heterojunction. During sliding, the superposition of the three electric fields is in the same direction as the built‐in electric field, resulting in a negative current. Similarly, the effect of applied bias voltage on the current of pGaN‐Si TVNG is studied (Figure 15b). Zhang et al. found a similar phenomenon in GaN and Bi_2_Te_3_ with different P/N types. When Bi_2_Te_3_ and GaN are separated, they exhibit different Fermi levels and surface states (Figure 15c). When two materials come into contact, electrons pass through the interface and transition between the two materials, generating an interfacial electric field (Figure 15d). During the rubbing, Bi_2_Te_3_ tends to lose electrons, while GaN gains electrons and stores them in surface states (defects and dislocations). The direction of the interfacial electric field is from Bi_2_Te_3_ to GaN, in the opposite direction of the built‐in electric field.^[^ ^34^ ^]^ The interfacial electric field plays a predominant role in the electrical output of this type of TVNG.

The interfacial electric field may contribute to the electrical output of various TVNG, and its direction is not necessarily aligned with the built‐in electric field. The direction of the total electric field superimposed by the two electric fields determines the direction of the TVNG electrical output.^[^ ^66^ ^]^ The interface electric field theory serves as a supplement to the previously recognized built‐in electric field and plays an important role in the power generation mechanism of TVNG.

## 5 Tribovoltaic Related Coupling Effect

As previously mentioned, the coupling effects are easy occurrence between tribovoltaic effect and other multi‐physics field effects, owing to the sensitivity of semiconductors to light, heat, force, magnetic, and electric fields. The coupling process makes it possible for a single semiconductor material to collect and perceive multiple sources of energy, while also potentially contains complex physical and chemical mechanism.

### 5.1 Tribo‐Photovoltaic Coupling Effect

**Figure 16:**
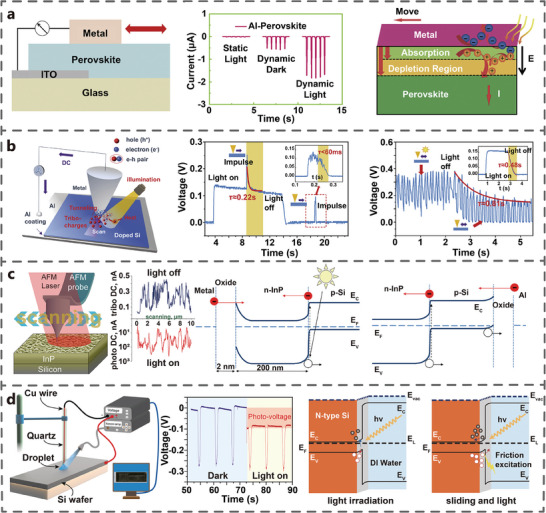
Tribo‐photovoltaic coupling effect. a) Schematic diagram of a generator with a metal/perovskite Schottky junction (Adapted from Hao et al.^[^ ^67^ ^]^ with permission. Copyright 2019, Elsevier B.V.). b) Schematic diagram of metal/silicon point contact system structure (Adapted from Liu et al.^[^ ^26^ ^]^ with permission. Copyright 2019, Elsevier B.V.). c) Schematic diagram of metal‐oxide‐semiconductor interface (Adapted under terms of the CC‐BY license.^[^ ^68^ ^]^ Copyright 2019, Sharov et al., published by American Chemical Society). d) Schematic diagram of interface diagram of DI water and Si crystal (Adapted from Zheng et al.^[^ ^69^ ^]^ with permission. Copyright 2021, Elsevier B.V.).

Hao et al. discovered the tribo‐photovoltaic coupling effect in a perovskite‐Al heterojunction TVNG for the first time (**Figure** **16**a). They found that the I_sc_ of TVNG under light conditions was three times higher than that in the dark, while the I_sc_ under static conditions was minimal. They believed that the heightened V_oc_ and I_sc_ were attributed to incident light inducing more photo‐generated carriers in the space charge region on the perovskite film surface.^[^ ^67^ ^]^ Meanwhile, Liu et al. also found a similar coupling phenomenon at the contact interface between stainless steel tip and pSi (Figure 16b). Under light conditions, the V_oc_ of TVNG can reach 0.6 V and the I_sc_ can reach 8 µA, which is much higher than the photocurrent without rubbing. When light illuminated the semiconductor surface, more electron‐hole pairs are excited by photons. The experiment found that the presence of light makes the decay time of the electrical output after stopping longer, which is caused by the significant slowing down of the electron‐hole pair recombination rate.^[^ ^26^ ^]^ Subsequently, Sharov et al. also observed the tribo‐photovoltaic coupling effect using metal tip and InP (Figure 16c). The peak current of TVNG is ≈1 µA. The dark current is only 0.5 nA, because electron hole pairs will be generated at the interface under laser irradiation conditions.^[^ ^68^ ^]^ Similarly, Zheng et al. discovered the tribo‐photovoltaic coupling effect at the liquid (DI water)‐semiconductor (Si) interface (Figure 16d). Under dark conditions, the V_oc_ of water‐silicon TVNG is −0.3 V. After light irradiation, the photocurrent is about −0.08 V.^[^ ^69^ ^]^ Compared with the dark state, the tribovoltaic signals have a little decrease. The increase in carrier concentration under light irradiation condition contributed to the enhancement of the electrical output, as depicted in the band diagram (Figure 16d).

Compared with single semiconductor effect, the coupling of light and friction can effectively improve electrical output and energy utilization efficiency.^[^ ^67^ ^]^ The new energy technology based on the tribo‐photovoltaic coupling effect represent a highly promising approach for achieving mechanical and solar energy harvesting in the future.

### 5.2 Tribo‐Thermoelectric Coupling Effect

**Figure 17:**
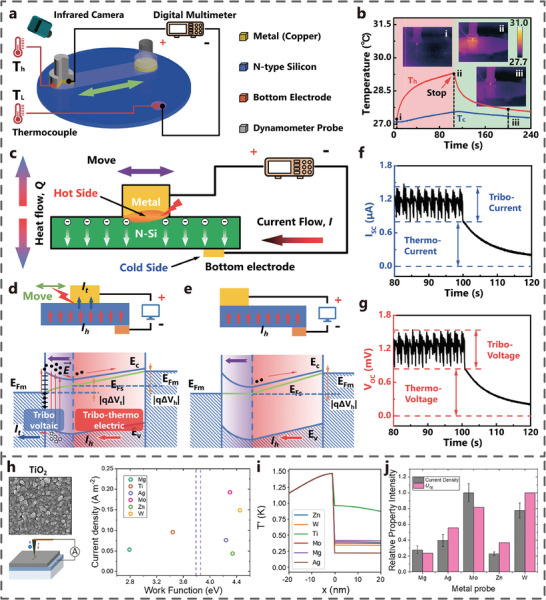
Tribo‐thermoelectric coupling effect. a) A 3D schematic for the experiment setup and structure of the TVNG. b) Temperature difference between hot side and cold side. c) Working mechanism of the tribo‐thermoelectric effect on MS interface. Working status and energy band diagram of n‐type silicon and metal (W_s_ < W_m_) d) in rubbing. e) after rubbing. f) Short‐circuit current output and g) Open‐circuit voltage output of the TVNG under a steady state. (Adapted from Zhang et al.^[^ ^28^ ^]^ with permission. Copyright 2021, Elsevier B.V.) h) Schematic image of the TiO_2_‐based TVNG and measured current density plotted against the material work function. i) Respective average temperature difference distributions. j) Normalized Seebeck effect voltage. (Adapted from Šutka* et al.^[^ ^70^ ^]^ Copyright 2023 American Chemical Society).

In addition to tribovoltaic effect, frictional heating can also generate thermoelectric effect in semiconductors. The coupling mode and mechanism of these two effects directly affect the output performance of the TVNG.^[^ ^28^ ^]^ Zhang et al. first studied the coupling effect of tribo‐thermoelectric and tribovoltaic by rubbing a copper on a silicon wafer (**Figure** **17**a). The friction energy excites electron‐hole pairs at the interface, leading to a direct current under the action of built‐in electric field.^[^ ^28^ ^]^ Simultaneously, the friction heat and the ununiform temperature distribution leads the major carriers to flow from the hot side to the cold side in the semiconductor (Figure 17b). The results show that the DC output of the metal‐semiconductor TVNG includes two parts: the stable part of the tribo‐thermoelectric effect and the fluctuating part of the tribovoltaic effect (Figure 17c,d).

The mechanism of the coupling effect can be explained by the semiconductor band diagram. Taking copper and nSi as an example, when the copper block and the semiconductor are separated, they have their own band structures and Fermi levels. When they come into contact, due to the different Fermi levels, electrons flow from the semiconductor to the copper block, and the band bends upward to form a built‐in electric field. During the rubbing process, a tribo‐current (I_t_) is generated due to the tribovoltaic effect, and a thermo‐current (I_h_) is also generated due to the thermoelectric effect of temperature gradient caused by frictional heating. Their directions are the same, thus they are positively superimposed (Figure 17e). When the rubbing stops, the current generated by the tribovoltaic effect immediately disappears, while the temperature gradient gradually dissipates, so there will still be a thermo‐current (I_h_) for a period of time (Figure 17f).

It was observed that a faster velocity, larger normal force can enhance the thermo‐voltage/current with larger temperature difference; the greater frictional energy is obtained, the larger tribo‐voltage/current can be formed. The four superimposed states of thermoelectric voltage/current and tribovoltaic voltage/current were theoretically and experimentally analyzed, and these superimposed states were proved to be jointly determined by the direction of the built‐in electric field and the majority carrier of the semiconductor (Figure 17g). The tribovoltaic effect is an interface effect closely related to the built‐in electric field formed at the interface. The tribo‐thermoelectric effect of frictional heating is a bulk effect closely related to the temperature difference inside the material. Compared with the instantaneous tribovoltaic effect, the tribo‐thermoelectric effect has an accumulating effect. In addition, Šutka et al. studied TVNG based on the metal‐TiO_2_ interface and found that the current density of TVNG had little relationship with the work function of metals (Figure 17h). Considering the influence of other physical effects on the metal‐semiconductor interface, the thermoelectric coefficients of different metals are tested (Figure 17i). The results show that there is a significant correlation between the thermoelectric coefficients and the current density of TVNG, among which the current density of molybdenum is the highest, reaching 192 mA cm^−2^ (Figure 17j), which provides a new idea for understanding the coupling mechanism of tribo‐thermoelectric effect.^[^ ^70^ ^]^

By studying the multi‐physical field coupling effect of friction and semiconductor, it can further promote the multi‐energy harvesting and sensing based on semiconductor interfaces.

## 6 Applications of TVNG

Although the tribovoltaic effect is a newly discovered semiconductor effect in recent years, it has already found applications in various fields, particularly in power supply and sensing. Compared with TENG, TVNG exhibits broader application prospects for self‐powered systems due to its advantages of DC electrical output, high current, and low impedance.^[^ ^27^ ^]^ This section provides an overview of the application cases of various types of TVNG developed thus far in the fields of energy harvesting and sensing.

### 6.1 Energy Harvesting Device and Application

**Figure 18:**
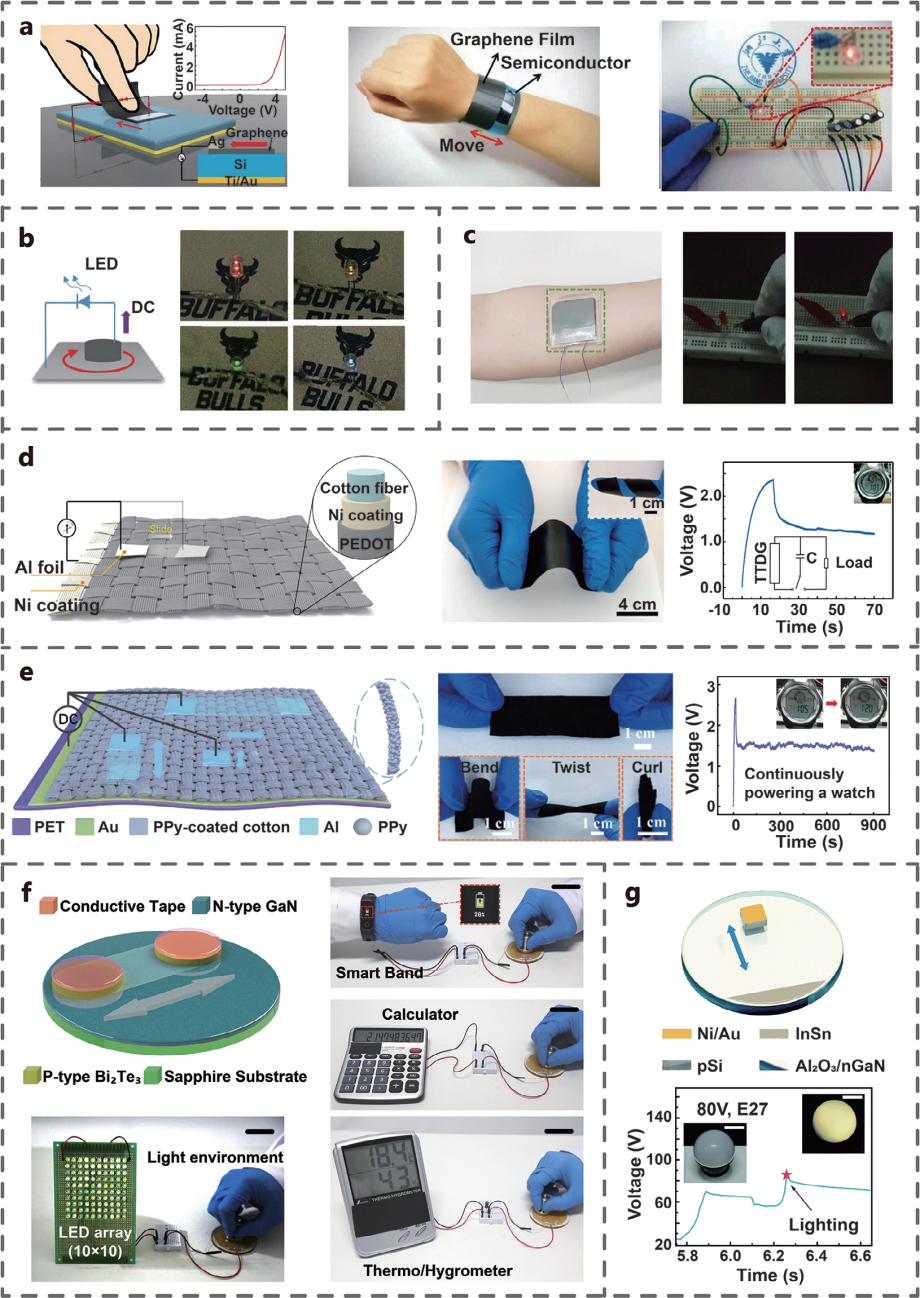
Energy harvesting device and application. a) An LED was powered by a capacitor charging from a Al/Si TVNG. (Adapted from Lin et al.^[^ ^58^ ^]^ with permission. Copyright 2018, WILEY‐VCH Verlag GmbH & Co. KGaA, Weinheim). b) Powering the LED bulb with the carbon aerogel/Si TVNG. (Adapted from Huang et al.^[^ ^85^ ^]^ with permission. Copyright 2022, WILEY‐VCH Verlag GmbH & Co. KGaA, Weinheim). c) An red LED driven by 6 FLGs in series. (Adapted from Liu et al.^[^ ^47^ ^]^ with permission. Copyright 2019, WILEY‐VCH Verlag GmbH & Co. KGaA, Weinheim) power the leds. d) A digital electronic watch was powered continuously by the seven flexible TVNGs. (Adapted under terms of the CC‐BY license.^[^ ^31^ ^]^ Copyright 2021, Meng et al., published by American Chemical Society). e) A commercial watch constantly powered by the TVNG. (Adapted from Meng et al.^[^ ^53^ ^]^ with permission. Copyright 2022, The Royal Society of Chemistry). f) Power supply for commercial electronic wristband, hygrometer, etc. (Adapted from Zhang et al.^[^ ^34^ ^]^ with permission. Copyright 2022, WILEY‐VCH Verlag GmbH & Co. KGaA, Weinheim). g) Driving high‐voltage light bulbs (Adapted from Wang et al.^[^ ^35^ ^]^ with permission. Copyright 2022, The Royal Society of Chemistry).

In the early study, TVNG was used as a power supply unit for low‐power devices such as LEDs. Lin et al. developed a TVNG based on graphene and silicon, which can generate a voltage output of 0.4 V. A series of five such TVNGs can light up a LED without a rectifier bridge. Meanwhile, a flexible wristband was developed using the TVNG based on flexible graphene film (20 µm) and GaAs film (2 µm) can generate DC output, which showcases the potential for wearable flexible energy harvesting and sensor devices (**Figure** **18**a). With the development of technology, the capacity of a single TVNG to power LEDs has been achieved.^[^ ^58^ ^]^ For example, Liu et al. developed a TVNG composed of carbon aerogel and silicon capable of directly illuminating LEDs of different colors (Figure 18b).^[^ ^47^ ^]^ Huang et al. connected six flexible TVNGs in series to power red LEDs. This TVNG was prepared by sputtering metal and semiconductor films on a polyethylene naphthalate (PEN) substrate, skin surface for human energy harvesting (Figure 18c). Flexible high‐power TVNGs have also been developed. For example, Pu et al. developed a TVNG composed of flexible semiconductor and Al, which can generate a V_oc_ of 0.4 V and an I_sc_ of 2.5 µA, and can power commercial electronic watches (Figure 18d). The flexible semiconductor is prepared by coating PEDOT on a textile substrate with Ni coating. This flexible TVNG can maintain stable electrical output after experiencing 3000 sliding or 6000 bending.^[^ ^31^ ^]^ To further improve the performance of TVNG, they also developed a TVNG composed of Al and fabric coated with PPy material. Its V_oc_ and I_sc_ can reach 1 V and 50 µA, respectively, and can remain unchanged after experiencing 8000 sliding and 10 000 bending (Figure 18e).^[^ ^53^ ^]^ In recent years, third‐generation semiconductors have emerged in many fields due to their unique properties of wide bandgap. For example, Zhang et al. developed a TVNG composed of GaN and Bi_2_Te_3_.The TVNG can generate a V_oc_ of 40 V and a power density of 11.85 W m^−2^, which are 6.5 times and 200 times that of TVNGs of the same magnitude before. Leveraging its high‐power characteristics, it can light up 100 LEDs and drive commercial electronic devices such as temperature and humidity meters, commercial smart bracelets, calculators, and commercial smart watches (Figure 18f).^[^ ^34^ ^]^ Wang et al. developed a TVNG based on GaN and Si that generates up to a V_oc_ of 130 V and a peak power density of 2.8 W m^−2^. It has the capability to drive a commercial bulb with a working voltage of 80 V, showcasing its potential for broader applications in mainstream electronic devices.^[^ ^35^ ^]^

The evolution of TVNG technology is evident from its humble beginnings, initially capable of lighting up only one LED, to the latest developments powering hundreds of LEDs.^[^ ^47^ ^]^ The transition to fabric‐based TVNG further expanded their capabilities, enabling the power supply for commercial electronic watches, which was a significant improvement. In recent years, high‐power TVNGs have been developed through research on third‐generation wide‐bandgap semiconductors. They can even power commercial temperature and humidity meters, commercial smart bracelets, and hundreds of LEDs.^[^ ^34^ ^]^ From the initial one LED to now hundreds of LEDs, this is a leap forward for the development of TVNG. Further improvements in material interfaces in the future will further improve the power generation performance and energy conversion efficiency of TVNGs. In addition, developing an adapted power management module will make TVNGs more market‐oriented and bring brighter application prospects for TVNGs.

### 6.2 Sensing Device and Application

**Figure 19:**
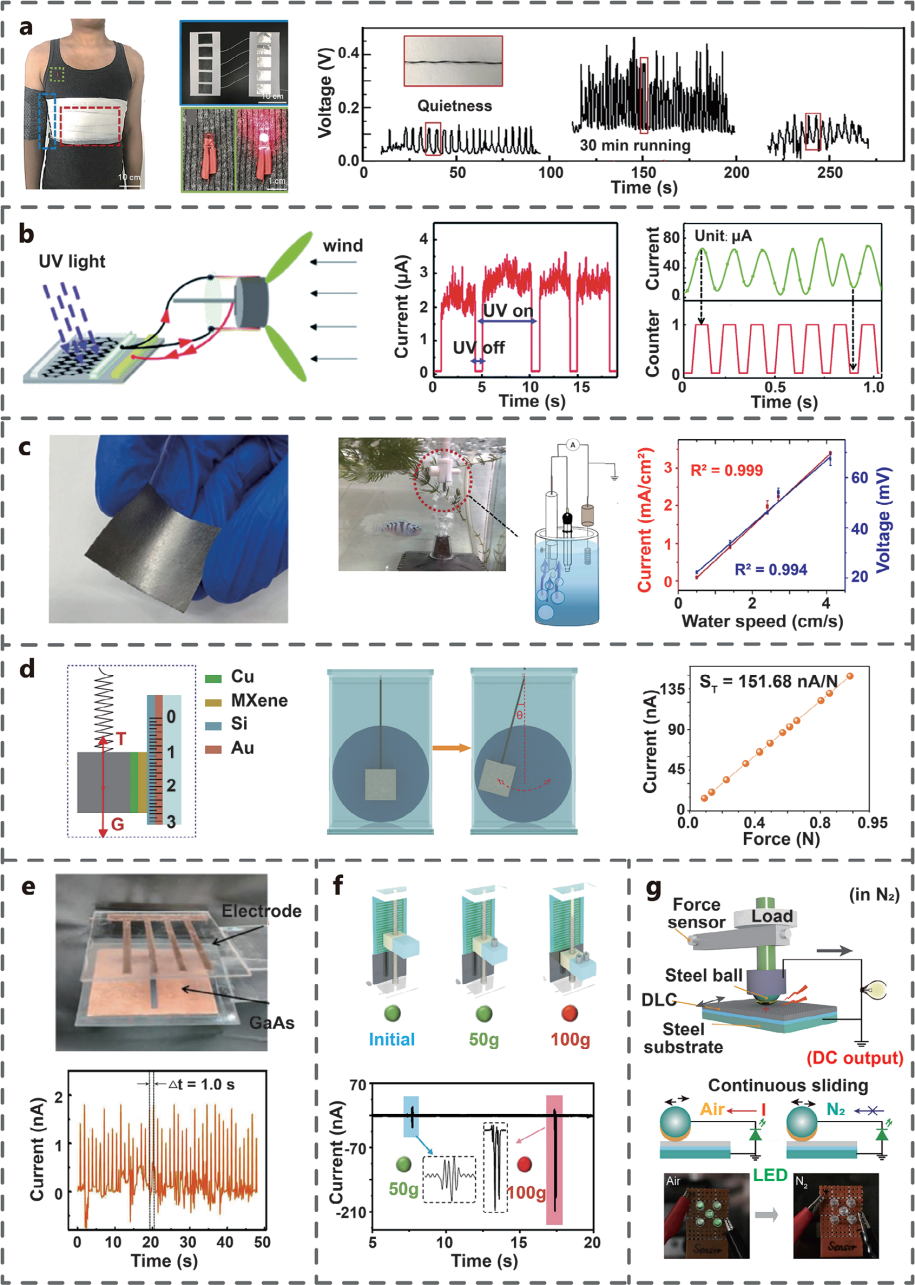
Sensing device and application. a) Respiratory monitoring (Adapted from Chen et al.^[^ ^52^ ^]^ with permission. Copyright 2021 Elsevier B.V.). b) Revolution record (Adapted from Huang et al.^[^ ^50^ ^]^ with permission. Copyright 2022 Elsevier B.V.). c) Self‐powered measurement of water velocity (Adapted under terms of the CC‐BY license.^[^ ^50^ ^]^ Copyright 2021, Yu et al., published by Royal Society of Chemistry). d) Sensing of displacement, rotation Angle and force (Adapted from Luo et al.^[^ ^43^ ^]^ with permission. Copyright 2022, WILEY‐VCH Verlag GmbH & Co. KGaA, Weinheim). e) Micro‐nano network displacement sensor (Adapted from Wang et al.^[^ ^71^ ^]^ with permission. Copyright 2023, WILEY‐VCH Verlag GmbH & Co. KGaA, Weinheim). f) Self‐powered weight sensing (Adapted from Qiao et al.^[^ ^36^ ^]^ with permission. Copyright 2022, WILEY‐VCH Verlag GmbH & Co. KGaA, Weinheim). g) Identification gas sensing applications under macro‐superlubric conditions (Adapted from Zhang et al.^[^ ^61^ ^]^ with permission. Copyright 2022 Elsevier B.V.).

**Figure 20:**
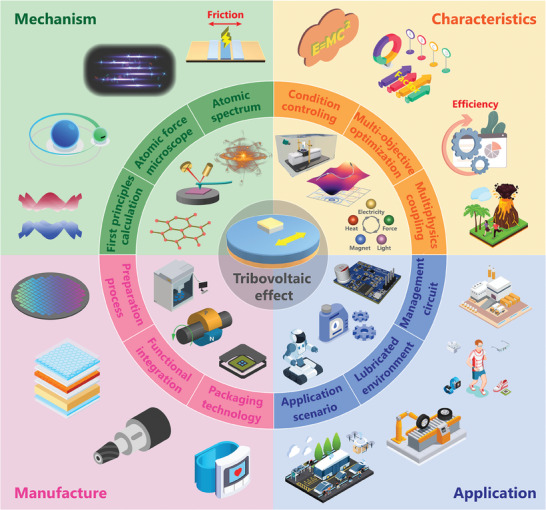
Four directions for the future development of the tribovoltaic effect.

TVNG exhibit versatile applications in various sensing fields. The flexibility of TVNGs makes them suitable for monitoring human body conditions and water flow. Chen et al. proposed a TVNG based on graphene oxide and aluminum capable of converting mechanical energy from chest movements during human breathing into electrical energy. This flexible TVNG is employed for monitoring respiratory rates and sweat levels on the skin, as the electrical output is influenced by ambient humidity (**Figures** **19**a and **20**).^[^ ^52^ ^]^ Rigid TVNG has advantages in sensing parameters such as rotation speed, force, displacement, and angle. Lin et al. developed a wind‐driven TVNG that demonstrated an average I_sc_ of 4.4 mA in 740 s, which is 1000 times that of previous metal/semiconductor‐type TVNGs (Figure 19b). By analyzing the frequency of the peak current, the TVNG can sense the speed of wind blades, providing valuable information on wind speed. The current of this TVNG composed of Cu and pSi is pulsed, so they use the frequency of the peak current of the TVNG to reflect the speed of the wind blades, thus achieving sensing of wind speed and other information.^[^ ^59^ ^]^ Huang et al. immersed a TVNG designed with fluorinated graphite paper in water to collect energy from water flow, and monitored water speed by the current of the TVNG (Figure 19c).^[^ ^50^ ^]^ Zhu et al. found that the I_sc_ of TVNG composed of Si and MXene increased linearly with increasing sliding speed.^[^ ^43^ ^]^ Therefore, they used this TVNG for sensing force, speed, displacement, and angle through some structural designed (Figure 19d). Some scholars have also achieved measurements of micro‐ and nano‐ scales through smart structure design. Li et al. utilized the interval time between peak I_sc_ generated by rubbing between an interrupted metal array and gallium arsenide and the sliding speed of the material to calculate the distance moved by the material (Figure 19e).^[^ ^71^ ^]^ They fabricated a grid structure with electrode widths of 10 µm and electrode spacings of 100 µm on a glass substrate. When gallium arsenide slides on the electrodes at a speed of 0.2 mm s^−1^, the TVNG generates dense current pulses. Based on the above positioning mechanism, they successfully measured the displacement of micro/nano grid, which showed potential in micro‐scale displacement detection. In order to mitigate energy dissipation caused by interface wear and improve the energy conversion efficiency of TVNG, many scholars have developed TVNGs based on lubricated/superlubricated interfaces. For example, Wang et al. used water‐based graphene oxide as a lubricant to improve the life of TVNG based on Cu and Si (Figure 19f). They applied the TVNG to bridge vibration and cargo overweight monitoring.^[^ ^36^ ^]^ Furthermore, Wang et al. found that the I_sc_ of TVNG composed of DLC and stainless steel with a superlubrication state (COF < 0.01) is different when placed in different gas environments such as nitrogen and air (Figure 19g). Therefore, they used this TVNG to power LEDs and monitored the superlubrication state through the brightness of the LED.^[^ ^61^ ^]^

In summary, TVNGs have been applied in flexible wearable respiratory monitoring, detection of parameters such as force, displacement, speed, and angle of moving objects,^[^ ^43^ ^]^ measurement of micro/nano‐scale displacement,^[^ ^71^ ^]^ monitoring of object weight,^[^ ^36^ ^]^ and other aspects. Additionally, it has also been used to monitor water flow rate,^[^ ^50^ ^]^ wind speed,^[^ ^59^ ^]^ lubrication state, and gas environment.^[^ ^61^ ^]^ These diverse applications indicate the continuous developed and breakthroughs in TVNG‐based sensing devices, with the potential for broader application in various life and industrial scenarios in the future.

## 7 Summary and Outlook

Although the tribovoltaic effect has only been discovered for a few years, as a new energy technology, it has already demonstrated sufficient theoretical significance and application prospects. With further research, this paper proposes a series of issues and challenges in four aspects: mechanism, characteristics, manufacture, and application.

### 7.1 Mechanism

Friction is generally considered as an energy dissipation process, which has eight possible mechanisms during sliding: wear, molecular deformation, thermal effect, electronic effect, bonding, phonon effect, environmental and chemical effect.^[^ ^72^ ^]^ Some studies have suggested that the energy of the tribovoltaic effect may come from the breaking and forming of atomic bonds, which can excite charge carriers. Alternatively, local heating caused by mechanical motion can lead to strong electro‐acoustic coupling and promote the formation of electron‐hole pairs. Meanwhile, the significant energy dissipation during rubbing process can greatly affect the output of the tribovoltaic effect, with the output of the ultra‐smooth interface being several orders of magnitude lower than that of the ordinary interface. It can be seen that the friction of semiconductor interfaces involves multiple physical mechanisms, and the tribovoltaic effect may couple interface friction, atomic bonding, and phonon excitation, among other processes. Its theory is not yet complete, and the energy conversion mechanisms that lead to mechanical energy transfer and electron transition and charge carrier transport are not yet clear. Additionally, unlike the photovoltaic effect, the interface constantly refreshes during rubbing, and the tribovoltaic effect under complex friction interface changes cannot be explained simply by band theory.

The investigation of the mechanism of the tribovoltaic effect may require the use of terahertz spectroscopy. The interface photon emission and absorption spectra induced by triboelectrification can be detected. By comparing with standard atomic emission spectra, the discrete characteristic peaks in the interface atomic spectra induced by triboelectrification can be analyzed to clarify the specific physical processes of micro energy transfer such as electron transfer, electron transition, and electron emission at the interface. On the other hand, the microscopic frictional behavior of materials can be calculated by combining first‐principles and molecular dynamics methods. The microscopic processes of molecular collisions, chemical bond formation and breaking, phonon excitation, and lattice vibration in the relative sliding of semiconductor heterojunctions can be analyzed. By combining theory and experiments, the energy of electron‐hole pairs generated by the space charge region can be quantitatively analyzed, and the way of frictional energy dissipation and energy conversion process can be determined. Finally, a set of governing equations for the electromechanical energy conversion of the tribovoltaic effect can be proposed. These works may form a new set of methods for electromechanical energy conversion and lay a good foundation for the application research of the tribovoltaic effect.

### 7.2 Characteristics

The influence of various factors such as different semiconductor materials, interface structural properties, and mechanical input parameters on the triboelectric characteristics of semiconductor interfaces is highly complex. Generally, it is believed that a larger bandgap width of semiconductor materials, stronger interface charge capacity, and greater input friction force can result in higher power output for Triboelectric Nanogenerators (TVNGs). However, there is no accurate mathematical equation yet discovered to describe the relationship between the I_sc_ and V_oc_ of TVNGs. Unlike TENGs, which are energy conversion devices that provide intrinsic continuous output, TVNGs typically use average power density to represent their output performance. However, the power density of TVNGs can differ by 7–8 orders of magnitude for different sizes. The lack of evaluation standards for TVNGs hinders the accurate assessment of the output performance . Therefore, it is urgent to propose a new performance coefficient similar to the thermoelectric figure of merit (ZT) to guide the selection and structural design of TVNG materials. By further optimizing the optimization coefficient through multi‐objective optimization methods such as orthogonal design, response surface method, neural network, genetic algorithm, etc., guidance can be provided for TVNG material selection and structural design.

Furthermore, although there are already some semiconductor materials that can be used to manufacture TVNGs, the efficiency of these materials is quite low. For example, silicon‐based TVNG only has an efficiency of 10^−7^ (the electric work divided by the work of friction force), which falls far short of the requirements for practical applications (photovoltaic 24%,^[^ ^73^ ^]^ thermoelectric 15%^[^ ^74^ ^]^). Improving the output efficiency of TVNGs is another important issue that needs to be addressed for the development of the tribovoltaic effect in the future. In addition, the coupling of multi‐physics effects and the tribovoltaic effect is also a significant problem. During the process of triboelectric generation, the interaction of different physical field factors (such as temperature, light, magnetic field, lubricating environment, etc.) and extreme environmental conditions affects the power generation and efficiency. By exploring the interaction mechanisms between these physical field effects and the tribovoltaic effect, we can better understand the essence of the tribovoltaic effect and provide better support for its applications.

### 7.3 Manufacture

Currently, semiconductors such as silicon, gallium arsenide, and gallium nitride have been extensively studied as tribovoltaic materials, but there is little work discussing the preparation of these materials. Although the manufacturing processes for semiconductors such as silicon, such as the Czochralski method, the float‐zone method, and the directional solidification method, are already mature, these methods are only suitable for producing bulk materials such as silicon wafers and blocks. In traditional mechanical structures, it is difficult for these semiconductor materials to replace metal components that have strong pressure resistance and good ductility. Semiconductor thin‐film growth techniques such as electron beam evaporation, magnetron sputtering, chemical vapor deposition, and wet epitaxy are important technologies for the preparation of structurally integrated TVNG. Growing semiconductor thin films on metal materials presents great challenges. On the one hand, many mechanical parts are made of alloy materials, and it is difficult to form the lattice structure required by semiconductors on the growth surface. On the other hand, the thermal expansion coefficients of metals and semiconductors differ significantly, and the semiconductor thin films grown are prone to forming dislocations, leading to cracking and damage. Therefore, how to use semiconductor thin‐film technology to grow high‐quality, high‐performance tribovoltaic output materials is an important issue currently faced.

In addition, for traditional mechanical components and new wearable and implantable structures, designing structurally integrated TVNGs and studying the manufacturing process flow of high‐performance tribovoltaic devices is also an urgent problem that needs to be solved. For example, based on the rolling bearing structure, a semiconductor coating is prepared on the bearing raceway, and relative rolling and sliding between the metal ball and the semiconductor coating are used to achieve friction energy recovery. Independent self‐powered bearing devices require the design of two TVNGs in series with the same current output direction, so that the currents are superimposed. Manufacturing may require the use of multilayer structures to form a strong bonding force for the semiconductor thin film, with intermediate layers made of metals such as nickel and titanium. Various semiconductor thin films, including single‐crystal and polycrystalline films, can be manufactured using processes such as rapid annealing and crystallization to explore more affordable manufacturing processes. At the same time, it is necessary to test the electrical characteristics of semiconductor thin films such as Ohmic contacts, electrodes, and leads to ensure reliability. For some special devices, integrated packaging is also required to prevent environmental influences during use.

### 7.4 Application

Although many literatures have reported the application of TVNG as a micro/nano energy harvesting and sensor device, the scenarios and scope of these applications still lack in‐depth consideration. Especially in the field of environmental energy harvesting, where TVNG competes with TENG, thermoelectric generators, solar cells, etc., what are the characteristics and advantages (irreplaceability) of TVNG as an energy device? Generally, TVNG has a high current density and power density under a small contact area, which makes it more advantageous in scenarios of micro and nano‐scale contacts, such as the contact between bearing balls or probes and magnetic disks. Another issue related to the application is reliability. Although some research mentioned that wear problems can be solved by lubricants or superlubricity, the addition of these factors may lead to a weakening of tribovoltaic effect, making energy harvesting or sensing impossible. Another approach is to reduce wear by using hard materials, but these are usually ceramic materials that do not have semiconductor properties. Research on the durability and failure mechanism of TVNG will deepen our understanding of the tribovoltaic effect, help us find application scenarios for tribovoltaic devices, and provide guidance for their optimization in practical applications.

In addition, as an energy harvesting device, the output of TVNG will vary with external excitation, and energy management modules are still needed for supplying power to electrical devices. For the working mode and impedance characteristics of TVNG, corresponding circuits need to be designed. These methods differ from the traditional AC to DC conversion, Buck voltage reduction, energy storage module of TENG, over‐current/under‐voltage protection, anti‐reverse flow, maximum power point tracking, battery charging and discharging management module of solar cells. Customized management schemes need to be designed based on the output characteristics of TVNG. Finally, integrating TVNG into a wireless sensor network can solve various problems related to autonomous industrial equipment, intelligent traffic, human health monitoring, such as self‐powered wireless sensor network node construction, multi‐node data processing, fault intelligent recognition, health diagnosis, etc.^[^ ^52^, ^75^, ^76^ ^]^ By utilizing TVNG technology to achieve both friction energy harvesting and friction state sensing simultaneously, it promotes the self‐powered and intelligence of the industrial Internet of Things and the Body Area Network. Furthermore, TVNGs also can promote or catalyze some chemical reactions,^[^ ^77^ ^]^ and it also has potential applications in the degradation of organic materials and recycling of lithium‐ion batteries in future green and environmental fields.

### 7.5 Summary

In summary, this review sheds light on the origin, current status, challenges, and prospects of the tribovoltaic effect on semiconductor interface. Since the tribovoltaic effect was first discovered on the surface of molybdenum disulfide in 2018, it has attracted the attention of scholars as a technology with ultra‐high current density, low impedance, and DC characteristics that is different from traditional TENG. TVNGs based on the tribovoltaic effect have differentiated into a variety of structures: metal‐semiconductor, semiconductor‐semiconductor, metal‐insulator‐semiconductor, semiconductor ‐insulator‐semiconductor, solid‐liquid, and flexible interfaces. TVNGs with different interfaces also have different output characteristics, and have corresponding response laws to different working conditions and interface states. Although the current research on the mechanism of the tribovoltaic effect is still in its infancy, the mainstream mechanism explanations can be divided into two types: the carrier transport theory dominated by the built‐in electric field and the interface electric field. The friction excited carriers move directionally under the action of the two electric fields, generating direct current. In addition, the addition of multi‐physics field effects makes the tribovoltaic effect more complex, and the coupling effects of friction, light, and heat are not simply superposition effects, which may bring more abundant physical mechanisms and application potential. The diverse interface makes TVNG not only as a triboelectric energy harvesting device, but also as a sensor device in the human body and biology. Although the current work has made some progress in the mechanism, characteristics, manufacturing, and application, there is still a lack of systematic, comprehensive and in‐depth research on the tribovoltaic generation mechanism, evaluation indicators, and standardized manufacturing. These issues will eventually be resolved as new experimental designs and methods are implemented. The tribovoltaic effect will also realize the efficient recovery and utilization of friction energy, promote the development of the Internet of Things era, and become an important support for energy saving and carbon reduction under the background of carbon neutrality.

## Conflict of Interest

The authors declare no conflict of interest.

## Author Contributions

Z.Z. performed conceptualization, visualization, data curation, formal analysis, wrote the original draft, wrote, reviewed and edited; L. G. performed visualization, data curation, and wrote the original draft; R.L. performed visualization, and wrote the original draft; Y.F. performed visualization; J.C. wrote, reviewed and edited; C.Z. supervised, wrote, reviewed and edited.
